# The Alpha/Beta-Hydrolase Fold Superfamily in *Brassica napus:* Expression Profiles and Functional Implications of Clade-3 BnABH Proteins in Response to Abiotic Stress

**DOI:** 10.3390/ijms26104746

**Published:** 2025-05-15

**Authors:** Yahui Ding, Lianqiang Feng, Pu Li, Xindeng Yang, Muzi Li, Hanxuan Liu, Jiamin Xu, Jitong Zhang, Shouwu Sun, Xiaona Zhou, Wenfang Hao, Yanfeng Zhang, Chang-Gen Xie

**Affiliations:** 1 State Key Laboratory of Crop Stress Resistance and High-Efficiency Production, College of Life Sciences, Northwest A&F University, Yangling 712100, Chinajitong@nwafu.edu.cn (J.Z.); haowenfang@nwsuaf.edu.cn (W.H.); 2 Hybrid Rapeseed Research Centre of Shaanxi Province, Yangling 712100, China

**Keywords:** alpha/beta-hydrolase, ABH-fold, esterase/lipase, BnABH

## Abstract

Alpha/beta hydrolase (ABHs) fold esterase/lipase proteins represent a prominent family within the serine hydrolase (SH) superfamily that includes esterases and lipases and other catalytic and non-catalytic proteins. ABHs play crucial roles in both the fundamental and secondary metabolic pathways, including the synthesis and degradation of triacylglycerols (TAGs), key components of plant oils. Despite their importance in oil production, the *ABH* gene family in the oil crop *Brassica napus* has not been comprehensively analyzed. In the present study, we identified 777 *BnABH* genes in the *B. napus* cultivar ‘Zhongshuang 11’ (ZS11). Phylogenetic analysis categorized these *BnABH* genes into 10 distinct groups. Twenty-four BnABHs were identified through esterase activity staining and mass spectrometry, 11 of which were classified into clade C3. Examination of the gene and protein structures, expression patterns, and *cis*-elements of the *BnABHs* in clade C3 suggested diverse functional roles across different tissues and in response to various environmental stresses. In particular, *BnABH205* was highly induced by high temperatures. Subcellular localization analysis revealed that the BnABH205 protein was localized to the plastid. Further analysis revealed five haplotypes within the coding and 3′ untranslated regions of *BnABH205* that were significantly associated with seed oil content (SOC). Overall, this study provides a comprehensive understanding of BnABHs and introduces a robust methodology for identifying potential esterase/lipase genes that regulate seed oil content (SOC) in response to environmental hazards, especially heat waves during seed maturation.

## 1. Introduction

*Brassica napus* (*B. napus*; also known as rapeseed) is the second-largest oilseed crop in the world, following soybean. One of the most significant nutritional and economic characteristics of *B. napus* is its seed oil content (SOC), which constitutes 30–50% of the seed dry weight [1,2]. Improvement of yield and SOC are the primary goals for *B. napus* breeding [3,4]. SOC is mostly governed by the dynamic balance between triacylglycerol (TAG) production and breakdown during seed maturation [5,6]. Oil bodies are specialized structures for storing TAG and other lipids in oilseeds [6,7]. The formation of oil bodies is a core event during the mid-to-late maturation stage, involving precise regulation of fatty acid (FA) synthesis, TAG assembly, and TAG packaging into oil bodies [6,7]. The biochemistry of FA synthesis, TAG assembly, and TAG storage take place in the plastid, endoplasmic reticulum (ER), and cytoplasm, respectively [7]. TAG assembly in the plastid and ER is a finely tuned process that is heavily influenced by the activities of esterases and lipases [6].

In plants, various kinds of esterases and lipases have been identified [6,8,9,10,11]. Many of these lipases/esterases belong to the superfamily known as the serine hydrolases (SHs), which often contain a conserved Ser/His/Asp(Asn) catalytic triad [10]. The majority of SHs belong to the alpha/beta-hydrolase (ABH) fold superfamily [10,12]. ABHs are found in nearly all organisms, having key roles in many primary and secondary metabolic pathways [10,12]. ABHs are known as enzymes responsible for the hydrolysis of ester bonds [10], but recently the non-catalytic versatility of this superfamily has been expanded [10,13,14,15,16]. Some ABHs function as ligand receptors for strigolactone and karrikin in plants and for butenolides in *Bacillus subtilis* [10,13,14,15,16,17]. Other ABHs are involved in the accumulation of dolichols (Dols) [18], which are crucial for protein glycosylation.

The core ABH structure consists of eight β-strands (parallel or mixed) surrounded by α-helices, forming the classic α/β-hydrolase fold [10]. The catalytic triad is located at the loops between the β-sheet and the α-helices [10]. The N- and C-termini often contain variable regions (such as lipid-binding domains, transmembrane domains, or regulatory domains), conferring specificity and functional variation [10]. The ESTHER database (https://bioweb.supagro.inrae.fr/ESTHER/ accessed on 14 March 2023) facilitates analysis of proteins or protein domains belonging to the ABH superfamily [12]. A genome-wide survey in *Arabidopsis thaliana* identified 638 members of the ABH superfamily [10]. However, comprehensive annotation of the ABH superfamily in other organisms remain elusive, which may be attributed to low sequence identity among the superfamily members [12,19,20,21,22,23].

Environmental hazards are persistent challenges to the production of seed oil from the floral transition phase to seed filling [24]. Heat stress, particularly during seed ripening, significantly impacts various yield-related factors in rapeseed, including seeds per plant, seeds per pod, seed mass, and seed oil content (SOC) and composition [24,25,26,27]. A recent study by Huang et al. (2019) revealed that heat stress inhibits the accumulation of seed oil by disrupting photosynthesis and the BnWRI1 signaling pathway [26]. Interestingly, elevated temperatures during both the daytime and nighttime have adverse effects on the yield and fatty acid profile of seed oil in *B. napus* [24].

Although many α/β-fold serine hydrolases function in lipid metabolism, no comprehensive investigation of the ABH superfamily in *B. napus* has been completed. In the current study, a total of 777 ABH-like genes were identified from the genome of *B. napus* cultivar Zhongshuang 11 (ZS11) [28]. A comprehensive study of the *BnABH* gene superfamily was conducted to gain insights into the evolutionary and functional characteristics of BnABHs. The gene *BnABH205* was significantly associated with seed oil content (SOC). This study aims to facilitate the identification of ABH-type esterase/lipase candidates that contribute to lipid mobilization in response to environmental hazards, specifically heat waves during the seed maturation phase.

## 2. Results

### 2.1. Genome-Wide Identification and Characterization of BnABH Genes

**Figure 1:**
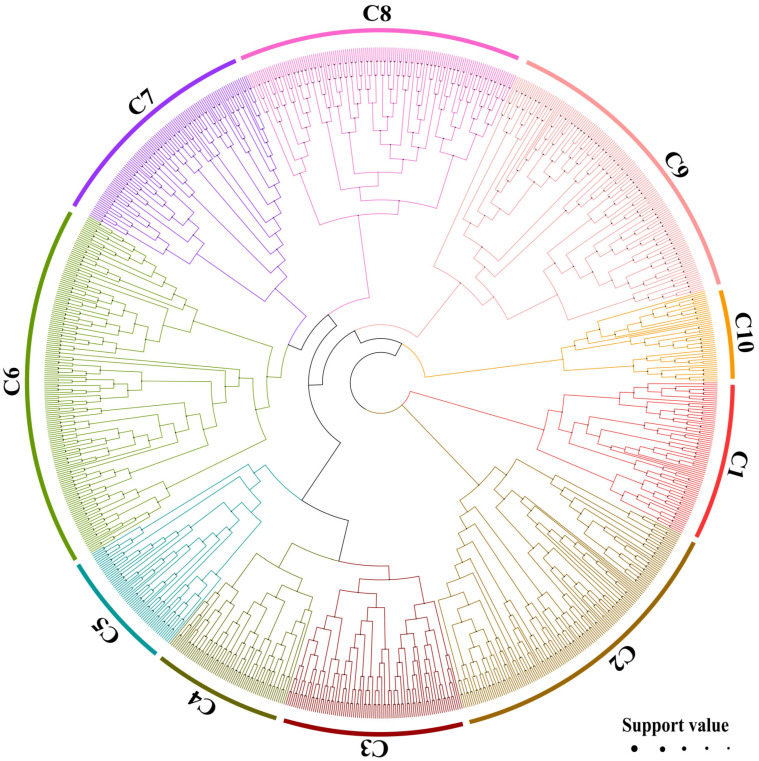
Phylogenetic analysis of AtABHs and BnABHs. Multiple sequence alignment of proteins was carried out using the FFT-NS-I algorithm implemented in MAFFT v7.490 software. FastTree v2.1.11 software was employed to construct phylogenetic trees. These proteins were clustered into 10 subclades, with the branches corresponding to different subclades marked with different colors. The size of the black dot indicates the nodal support.

A total of 777 putative BnABH genes were isolated across all 19 chromosomes of the *B. napus* cultivar ZS11. These genes were systematically redesignated as *BnABH1* through *BnABH777* based on their chromosomal locations. To assess the evolutionary relationships between ABH genes in *B. napus* and *A. thaliana*, we generated a phylogenetic tree utilizing the 777 full-length BnABH proteins alongside the 265 confirmed AtABH proteins. The NCBI Conserved Domain Database (CDD) and the SMART Tool were employed for validating AtABH proteins. As depicted in Figure 1, the BnABH proteins were grouped into 10 clades. The largest clade (C6) contained 142 proteins, while the smallest (C5) housed just 25 proteins. Within most subclades of the phylogenetic tree, *B. napus* had an expanded gene complement that was three or more times that of *A. thaliana* (Figure 1 and Figure S1). Subclade 5 was an exception, showing only a two-fold expansion (Tables S1 and S2). This divergence underscores the distinct evolutionary forces driving subclade-specific expansion dynamics.

The *BnABH* genes exhibited considerable variation in the number of introns, ranging from 0 introns in 46 *BnABH* genes to 55 introns in a single *BnABH* gene (*BnABH455*). On average, each *BnABH* gene contained seven introns (Table S1). The predicted amino acid (aa) sequences ranged from 54 residues (BnABH528) to 2655 residues (BnABH366), with a mean length of 519 residues (Table S1). The theoretical isoelectric point (pI) values of the BnABH proteins exhibited a wide range, from 4.31 (BnABH477) to 10.30 (BnABH533, Table S1). Evaluation of protein stability classified 505 BnABH proteins as unstable (with an instability index > 40) and 272 BnABH proteins as stable. The majority of the BnABH proteins (76.6%) were predicted to localize in either the chloroplast (255), cytoplasm (198), or nucleus (142). Remarkably, 79 BnABH proteins were predicted to localize to the plasma membrane, 13 to the ER, and a single protein (BnABH590) to the Golgi apparatus (Table S1).

### 2.2. Chromosome Distribution of BnABH Genes

**Figure 2:**
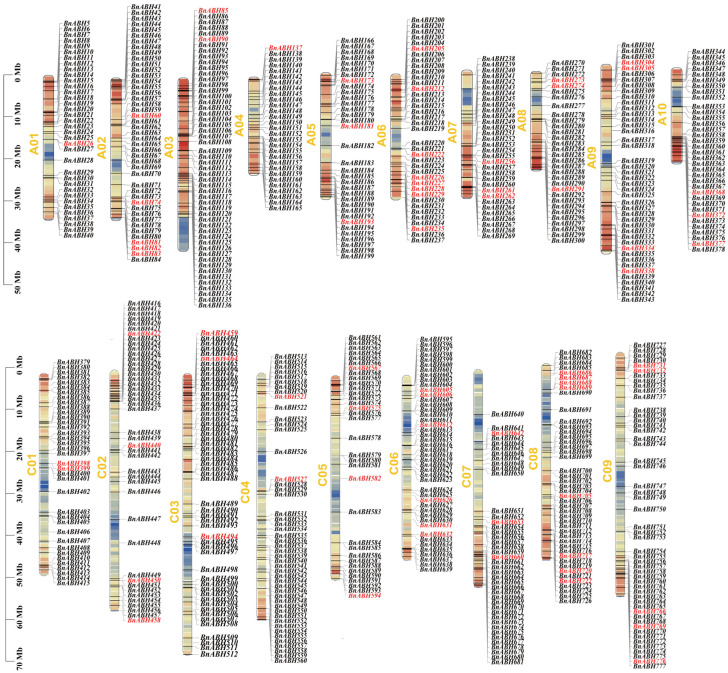
Distribution of the identified *BnABH* genes on the 19 chromosomes of *B. napus.* Chromosome numbers are indicated at the top of each bar and chromosomal distances are given in Mbp at the left of each bar. The *BnABH* genes from the C3 group are labeled in red; the other members of *BnABH* genes are labeled in black.

All of the *BnABH* genes were anchored on the chromosomes and depicted with MapChart [6]. Of the 777 predicted *BnABH* genes, 773 (99.48%) were mapped onto the 19 chromosomes of *B. napus* (Table S1, Figure 2). The *BnABH* genes were unevenly distributed across the 19 chromosomes of the A and C subgenomes, with 374 mapped to the A subgenome and 399 *BnABHs* to the C subgenome. In contrast, four *BnABH* genes remained on unmapped scaffolds. Higher *BnABH* gene densities were noted for chromosomes C03 (with 54 genes), A03 (52 genes), and C09 (51 genes), whereas chromosomes A04 (with 29 genes), A08 (with 31 genes), and A07 (with 32 genes) had fewer *ABH* family members (Figure 2).

### 2.3. Gene Duplication and Collinearity Survey of the BnABH Genes

**Figure 3:**
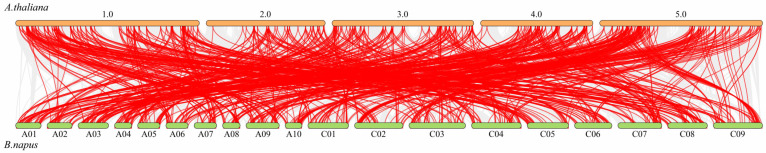
Synteny evaluation of *AtABH* and *BnABH* genes. The gray lines denote all synteny blocks, while the red lines depict orthologous relationships between *AtABH* and *BnABH* genes identified via synteny analysis.

Gene duplication events are a major driving force behind the expansion of gene families, a process closely linked to the evolutionary trajectory of plant genomes [28]. To understand the evolutionary relationships among *BnABH* genes, we conducted a thorough synteny analysis between the *B. napus* and *A. thaliana* genomes using MCScanX plugin within TBtools v2.210 [6,29]. This analysis identified 776 collinear gene pairs across the two genomes (Figure 3 and Table S3). Most *AtABH* genes have multiple orthologous genes in *B. napus*. For instance, three *AtABH* genes (AT1G52695, AT2G24320, AT4G31020) correspond to ten *BnABH* genes, indicating the expansion of the gene family in rapeseed (Table S3). A genome-wide syntenic analysis of the *BnABH* gene family in *B. napus* revealed 1024 syntenic gene pairs, categorized into two duplication modes: 15 tandem duplications (1.5%) and 1009 segmental duplications (98.5%) (Figure S2 and Table S4). This pattern may be strongly related to the polyploidization events during *B. napus* evolution, which led to extensive genomic replication and recombination, a conducive environment for gene family expansion. Further analysis showed an uneven distribution of duplication events between the A and C subgenomes: 189 in the A subgenome and 183 in the C subgenome. Inter-subgenomic duplication events were most prevalent, totaling 652 (Table S4). The syntenic network revealed complex evolutionary mechanisms, including multi-to-multi and single-to-multi gene relationships, suggesting that multiple evolutionary processes have formed the current *BnABH* gene family.

### 2.4. Eleven Members of the Clade-C3 BnABH Protein Group Are Mainly Related to Esterase Isozyme Activity During Seed Maturation

**Figure 4:**
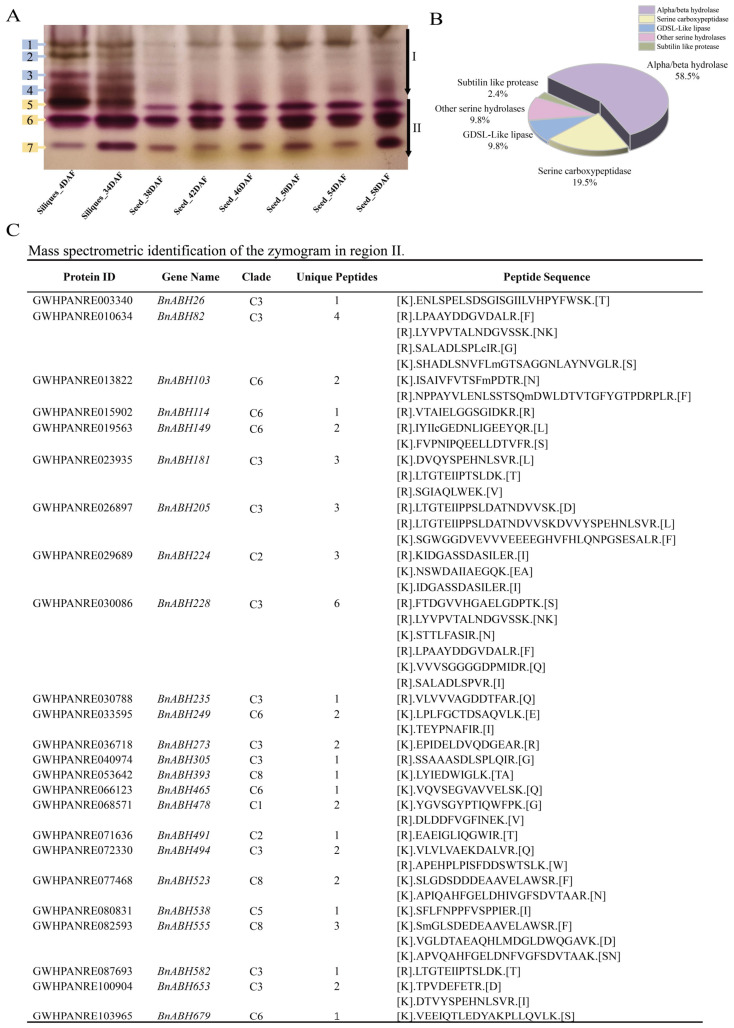
Zymogram of esterase isozymes isolated from siliques of *B. napus* cultivar ZS11 at different stages. (**A**) Zymogram of esterase isozymes in ZS11 siliques at different stages. Developmental stages of siliques or seeds are labeled below, with zymogram bands numbered from top to bottom on the left side. (**B**) Distribution of the 30 identified esterases under various classes. The identified protein portfolio represents different classes of SHs (ABH-type esterases, GDSL lipases, serine carboxypeptidases, subtilisin like protease, and other SHs). (**C**) Mass spectrometric identification of different bands in region II of the zymogram in (**A**).

To investigate the esterases that contribute to SOC during seed maturation, total protein extracts from siliques at various developmental stages were separated by polyacrylamide gel electrophoresis (PAGE). Following staining, zymogram differences were analyzed, and esterase isozymes were categorized into two regions (I and II) based on their band mobility (Figure 4A). During the early stages of silique development (4 DAF and 34 DAF), esterase activities in both regions of the zymogram were relatively high (Figure 4A). However, as the seeds matured (38 DAF onwards), enzyme activity in region I gradually decreased or disappeared, whereas region II bands maintained consistent activity levels. Within region II, the fifth and sixth bands exhibited stronger intensity compared to the seventh band (Figure 4A). These observations suggested that the esterase isozymes in region II may play a sustained role in regulating SOC during the critical period of oil accumulation.

To identify the esterase isozymes primarily responsible for the activity in zymogram region II, three enzyme bands from this region were excised and subjected to mass spectrometry analysis. As depicted in Figure 4B, we identified 41 SH esterases, which included 24 ABH-type esterases, 8 serine carboxypeptidases, 4 GDSL lipolytic enzymes, 1 subtilisin-like protease, and 4 unclassified SHs. Notably, ABH-type esterases predominated, constituting 58.5% of the total and significantly outnumbering other types (Table S5). Our phylogenetic analysis, presented in Figure 1, revealed that these esterases were distributed across several clades: C1 (BnABH478), C2 (BnABH224 and 491), C3 (encompassing BnABH26, 82, 181, 205, 228, 235, 273, 305, 494, 582, and 653), C5 (BnABH538), C6 (BnABH103, 114, 149, 249, 465, and 679), and C8 (BnABH393, 523, and 555). Approximately 45.8% of the identified esterases belonged to clade C3 (Figure 4C), indicating a potential specificity for these esterases in SOC regulation during the later stages of seed maturation.

### 2.5. Gene Structure and Conserved Motif Analysis of BnABHs in Clade C3

**Figure 5:**
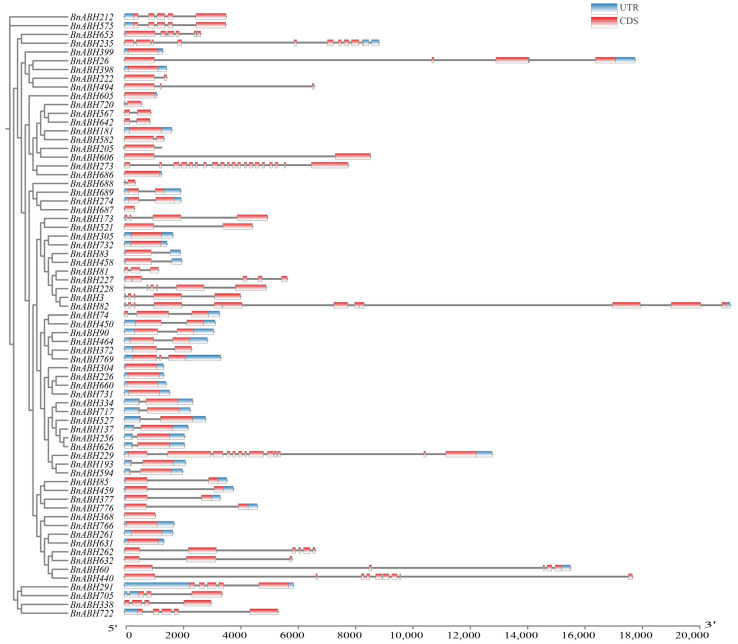
Schematic exon/intron structures of the clade-C3 *BnABH* genes. The red boxes denote exons, while the black lines represent introns. Blue boxes indicate untranslated regions (UTRs). The scale at the bottom allows estimation of the length of each coding sequence (CDS).

We focused on the BnABH proteins found in the three bands of region II of the zymogram (Figure 4) and decided to systematically analyze the proteins within clade C3. An examination of the exon/intron profiles revealed that about 40% of the *BnABH* genes (28 out of 69) in this clade had at least one intron. *BnABH273* had the highest number of introns (19), while 16 genes were completely devoid of introns (Figure 5). In the *BnABH205* subclade, only 20% of the members (2 out of 10) exhibited unique exon/intron structures (Figure 5). Within the entire C3 subfamily, protein motif conservation analysis showed that most members of the same cluster shared similar motif arrangements (Figure 5, Figures S3 and S4 and Table S6). Additionally, we investigated the protein architecture of the eleven proteins in clade C3 identified from the zymogram. Consistent with the gene structure findings, only the homologous proteins within the same subclade had comparable motif arrangements (Figure 5 and Figure S4). We also predicted the three-dimensional structures of proteins in the C3 clade, including twenty AtABHs and nine paralogs of BnABH205 (83, 205, 228, 273, 305, 458, 606, 686, 732) using the AlphaFold3 online tool (Figures S5 and S6). These homologs shared a highly conserved overall architecture, primarily comprising α-helices, β-sheets, and minor loop structures. Their core structures consisted of eight β-strands surrounded by α-helices (Figures S5 and S6). AT1G49650 displayed high structural similarity to BnABH205 (RMSD = 0.301) (Figure S5A), whereas AT5G62180 showed the greatest structural alignment with BnABH305 (RMSD = 0.236) (Figure S5B). Notably, some proteins exhibited distinct differences in loop regions and terminal structures, which may influence protein stability, interaction capabilities, or biological functions (Figures S5 and S6).

### 2.6. Expression Patterns of BnABH Genes in Clade C3

**Figure 6:**
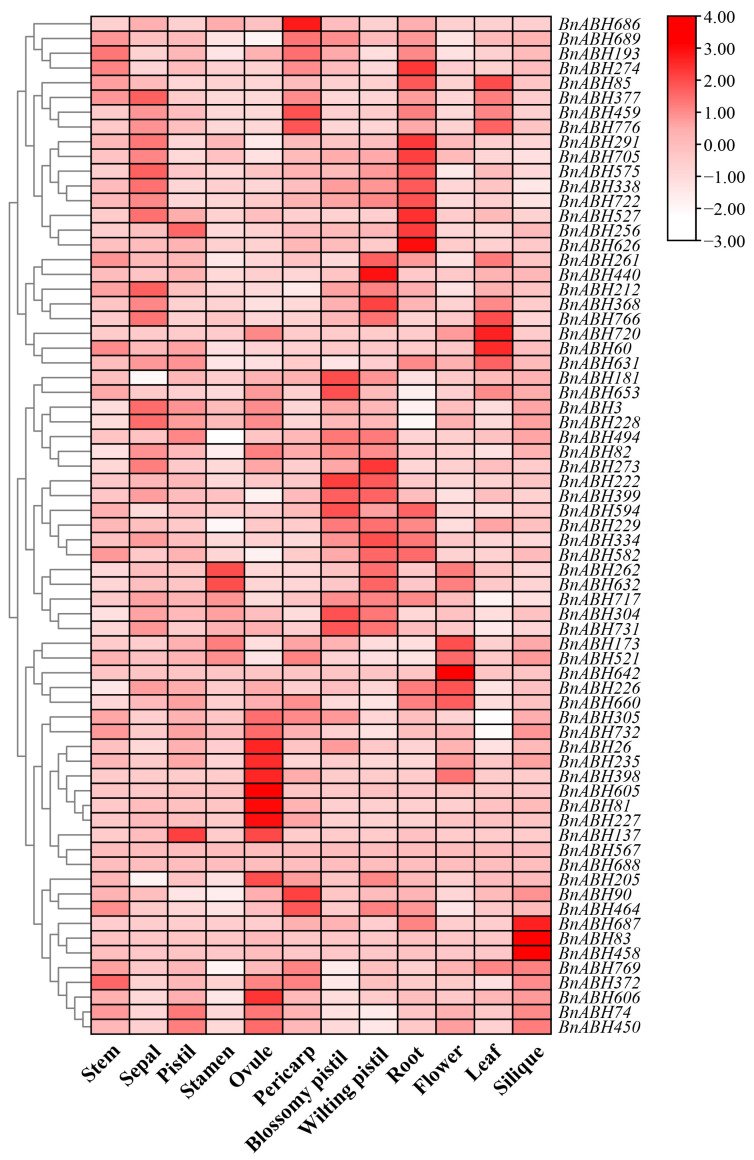
Expression profiles of the clade-C3 *BnABH* genes in different tissues. RNA-seq data corresponding to 12 distinct tissues (root, stem, leaf, flower, silique, sepal, pistil, stamen, ovule, pericarp, wilting pistil, and blossomy pistil) of the *B. napus* cultivar ZS11 were retrieved from publicly available transcriptome datasets. Subsequently, the transcripts per million (TPM) values were subjected to a log2 transformation followed by row-wise clustering. The color gradient depicted in the heatmap represents the relative expression levels, spanning from low (white colored) to high (red colored).

Tissue-specific expression profiles of genes provide crucial insights into their biological roles [6,29,30]. We utilized publicly available transcriptome data from *B. napus* ZS11 to extract expression data for 69 *BnABH* genes from clade C3 across various tissues. We generated heatmaps to visualize their expression patterns. Only two genes were undetected in all tissues analyzed. Our results showed that these *BnABH* genes exhibited distinct expression patterns in different tissues (Figure 6 and Figure S7). For instance, *BnABH722* and *BnABH705* had extremely high expression levels in roots. In floral tissues, genes like *BnABH521*, *BnABH226*, and *BnABH222* exhibited the highest expression. Additionally, some genes, such as *BnABH222*, *BnABH494*, and *BnABH228*, showed the highest expression levels in siliques.

The expression levels of the eleven C3 *BnABH* genes encoding proteins isolated in the zymogram (*BnABH26*, *82*, *181*, *205*, *228*, *235*, *273*, *305*, *494*, *582*, and *653*) exhibited considerable variation. Notably, *BnABH494* exhibited widespread and significantly high expression across tissues, while *BnABH26* and *BnABH235* showed relatively high expression exclusively in the ovule, with minimal expression detected in other tissues. *BnABH228*, *BnABH82*, and *BnABH273* shared similar expression profiles, with elevated levels observed in sepals, ovules, and pistils. *BnABH205*, *BnABH181*, and *BnABH653* showed tissue specificity for ovules and blossom pistils. *BnABH305* and *BnABH582* maintained moderate expression levels across various tissues, including the ovule and pericarp (Figure 6 and Table S7). Remarkably, *BnABH205* stood out as the sole gene with high expression during the later stages of seed ripening, distinguishing it from the other ten C3 BnABH proteins identified via mass spectrometry (Figure S8). Collectively, these findings suggested that the *BnABH* gene family exhibits tissue-specific expression patterns and may have undergone functional differentiation in *B. napus*.

### 2.7. Expression Profiles of the Clade-C3 BnABH Genes in Response to Abiotic Stresses

**Figure 7:**
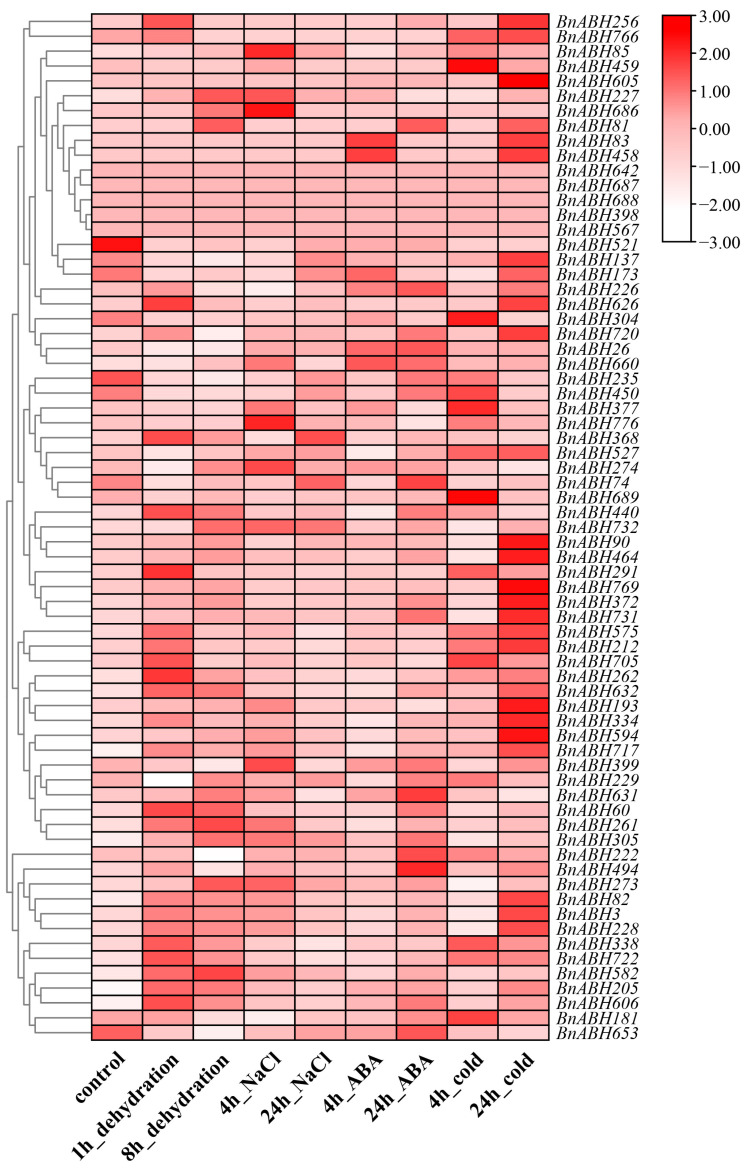
Expression profiles of the clade-C3 *BnABH* genes under different stresses. RNA-seq data generated from the plants of *B. napus* cultivar ZS11 grown under diverse stress conditions, namely dehydration, NaCl, abscisic acid (ABA), and cold stress, were retrieved from publicly available transcriptome datasets. Subsequently, the transcripts per million (TPM) values were subjected to a log2 transformation followed by row-wise clustering. The color gradient depicted in the heatmap represents the relative expression levels, spanning from low (white colored) to high (red colored).

To investigate the influence of various environmental stresses on the expression levels of *BnABH* genes from the C3 subfamily, we retrieved expression data and generated heatmaps to visualize their expression profiles. Under all the environmental stresses examined, just five genes were undetected. Our analysis revealed that this *BnABH* gene subfamily displayed a wide array of expression patterns in response to different stress conditions (Figure 7 and Figure S9). Notably, dehydration significantly up-regulated *BnABH632*, *BnABH722*, and *BnABH717*, with expression levels increased >25-fold after 1 and 8 h of treatment. Similarly, NaCl treatment substantially up-regulated *BnABH717* and *BnABH205*, with over four-fold increases after 4 and 24 h of salinity treatment. Furthermore, *BnABH205* and *BnABH606* were markedly up-regulated, with expression levels more than five-fold higher after 4 and 24 h of ABA treatment. Additionally, cold treatment increased expression of *BnABH717*, *BnABH212*, and *BnABH722*, exceeding 20-fold after 4 and 24 h of cold. Conversely, certain genes were significantly down-regulated by specific treatments, including *BnABH235* and *BnABH653* by cold, *BnABH450*, *BnABH521*, and *BnABH235* by ABA, *BnABH521* and *BnABH235* by NaCl, and *BnABH235* and *BnABH304* by dehydration.

The expression patterns of the eleven zymogram-identified clade-C3 *BnABH* genes (*BnABH26*, *82*, *181*, *205*, *228*, *235*, *273*, *305*, *494*, *582*, and *653*) within clade C3 exhibited considerable variation in response to environmental stresses. Specifically, *BnABH82* was stimulated by all the applied treatments, whereas its closely related paralog, *BnABH228*, displayed significant induction exclusively after 1 h dehydration and 24 h cold treatment (Figure 7 and Table S8). Likewise, *BnABH653* expression was markedly suppressed by dehydration, NaCl, and cold treatments; however, its close paralog, *BnABH26*, showed significant repression only in response to dehydration treatment (Figure 7 and Table S8). *BnABH273* expression was enhanced by 8 h dehydration and 4 h NaCl treatments but experienced a slight reduction after 4 h cold treatment (Figure 7 and Table S8). Conversely, *BnABH181* expression was slightly increased by 4 h cold treatment, yet decreased in response to 8 h dehydration and 4 h NaCl treatment (Figure 7 and Table S8). Additionally, *BnABH26* expression was slightly elevated by 4 h ABA treatment but reduced following 1 h and 8 h dehydration treatments (Figure 7 and Table S8). *BnABH205* exhibited significantly increased expression levels under various abiotic stresses, whereas *BnABH235* was inhibited under these conditions. *BnABH582* showed a marked increase in expression under drought stress, and *BnABH494* maintained high expression levels across all stress treatments (Figure 7 and Table S8).

As previously documented, high temperatures during seed maturation pose challenges to the yield and fatty acid composition of *B. napus* seed oil [24,25,26,27]. To investigate the impact of heat stress on the expression behavior of the eleven zymogram-identified clade-C3 *BnABH* genes (*BnABH26*, *82*, *181*, *205*, *228*, *235*, *273*, *305*, *494*, *582*, and *653*), we collected expression data and generated heatmaps to depict their expression profiles. Notably, *BnABH82*, *205*, and *305* were substantially up-regulated, with expression levels more than doubling after 1 h of heat exposure (Figure S10 and Table S9). Furthermore, *BnABH305* exhibited similar expression trends following both drought and heat treatments (Figure S10). Conversely, expression of *BnABH181* and *273* slightly decreased in response to heat treatment (Figure S10 and Table S9). Collectively, the diverse expression profiles of these genes suggested that the eleven C3-clade BnABH proteins with in vitro esterase activity may serve various functions in response to environmental stresses.

### 2.8. Analysis of Cis-Elements in Promoters of the BnABH Genes in Clade C3

**Figure 8:**
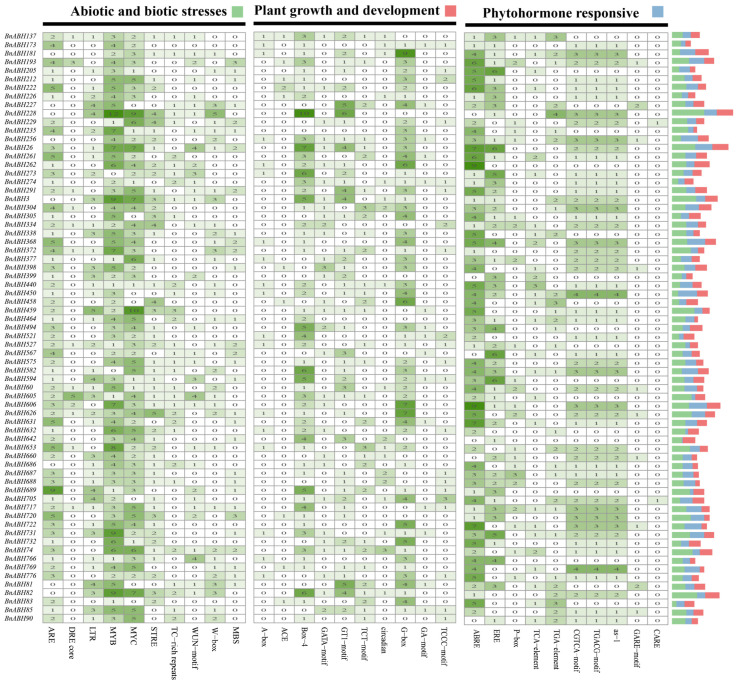
Predicted *cis*-elements in the promoter regions of the clade-C3 *BnABH* genes. The names of different *cis*-elements are shown at the bottom of the heatmap. These elements are classified into three main functional groups: abiotic and biotic stresses, phytohormone response, and plant growth and development. Each row represents a member of the gene family. The numbers in the cells of the heatmap indicate the quantity of each *cis*-element in the corresponding gene promoter. The darker the green color, the greater the quantity. The stack on the right-hand side further demonstrates the total numbers of different *cis*-elements in each functional category.

To gain insights into the expression behavior of the *BnABH* genes belonging to the C3 subfamily, we predicted the *cis*-acting elements in their promoter regions (Figure 8). Our analysis revealed the presence of 12 distinct *cis*-acting elements in the promoters of these genes, including numerous regulatory motifs associated with stress responses, plant growth and development, and phytohormone signaling (Figure 8 and Figure S11 and Tables S10 and S11). Notably, the majority of the C3-clade *BnABH* genes harbored *cis*-acting elements associated with abscisic acid responsiveness and anaerobic induction. Specifically, 63 genes contained abscisic acid responsive elements and 60 genes possessed elements linked to anaerobic induction (Figure 8 and Figure S11 and Tables S10 and S11). Furthermore, 52 genes featured *cis*-acting elements related to methyl jasmonate (MeJA) responsiveness, while 42 genes carried elements associated with low-temperature responses (Figure 8 and Figure S11 and Tables S10 and S11). The MYB binding site, which is associated with transcription factors involved in drought response and flavonoid biosynthesis, was identified in 29 and 6 genes, respectively. Additionally, we found *cis*-acting regulatory elements responsive to auxin (35 genes), gibberellin (40 genes), and salicylic acid (27 genes) in the promoter regions of the C3-clade *BnABH* genes. Remarkably, 11 out of the 12 *cis*-acting elements were present in the promoters of genes in clade C3 encoding the eleven BnABH proteins identified by mass spectrometry (*BnABH26*, *82*, *181*, *205*, *228*, *235*, *273*, *305*, *494*, *582*, and *653*) (Figure 8 and Figure S11 and Tables S10 and S11). The high abundance of *cis*-acting elements involved in responses to abscisic acid, MeJA, anaerobic induction, auxin, defense, and stress response in over half of the *BnABH* genes in the C3 clade suggests their potential roles in abiotic and biotic stress and phytohormone signaling pathways.

### 2.9. Subcellular Localization of BnABH205

**Figure 9:**
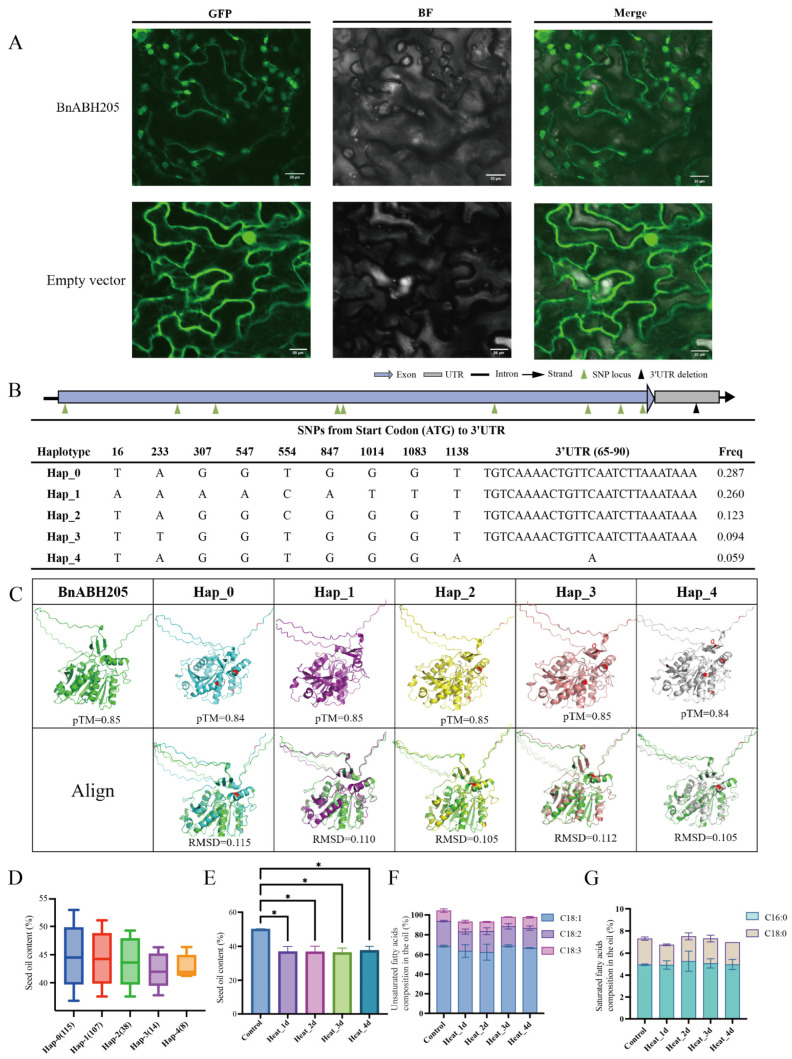
The subcellular localization and haplotypic variations of BnABH205 exhibit a notable association with SOC. (**A**) The BnABH205 protein was visualized using green fluorescent protein (GFP) fluorescence. The upper row displays the localization of BnABH205 protein, and the lower row serves as the empty vector control. GFP indicates the green fluorescent protein fluorescence signal. Scale bars represent 20 μm. (**B**) A diagram detailing the distribution of SNPs from the initiation codon (ATG) to the 3′ UTR and haplotype frequency. The allelic combinations of SNPs for five haplotypes (Hap_0 to Hap_4) are enumerated alongside their respective frequencies. (**C**) The structures of BnABH205 and its variant haplotype proteins (Hap_0 to Hap_4) are depicted in distinct colors (green, blue, purple, yellow, pink, and gray, respectively), with mutated amino acids highlighted in red. Protein alignments were performed using PyMOL 3.0.3 software, and the resulting RMSD values are indicated beneath each protein. (**D**) Differences in seed oil content were observed among the different haplotypes (Hap_0 to Hap_4). (**E**) Changes in SOC were assessed under various heat treatment conditions (Heat 1 d, Heat 3 d, Heat 4 d) and compared to the control group. (**F**) The ratios of unsaturated fatty acids (C18:1, C18:2, C18:3) in oil were examined under different heat treatment conditions. (**G**) Shifts in the composition of saturated fatty acids (C16:0, C18:0) in oil were observed under varying heat treatment conditions. Each experiment was repeated at least three times to ensure reliability. Error bars represent the standard deviation, and asterisks (* *p* < 0.05) are used to indicate statistically significant differences.

Based on the expression profiles of the eleven *BnABH* genes within clade C3 identified through mass spectrometry, *BnABH205* was selected for further study due to its significantly elevated expression levels during the later stages of seed maturation and its pronounced response to environmental stress (Figures S8 and S10). Determination of protein subcellular localization provides insights into the potential roles and interactions of a protein within the cellular environment. Initial predictions suggested that BnABH205 localizes to the cytoplasm (cytol, Table S1). A BnABH205-GFP fusion protein was generated and transiently expressed in tobacco leaves. The BnABH205-GFP fusion protein exhibited primary fluorescence in the plastid (Figure 9A), deviating from the initial prediction. A faint green fluorescence of BnABH205-GFP was also observed in membrane-like structures, hinting at a potential association with these compartments. Collectively, these findings imply that BnABH205, functioning as an esterase, may play a role in TAG synthesis within the plastid during seed maturation.

### 2.10. Five Haplotypes of BnABH205 Exhibit Correlation with SOC

The production of oil in seeds hinges on the coordinated metabolic pathways of the de novo synthesis of fatty acids in plastids (which differentiate into proplastids during seed maturation) and assembly of TAGs in the cytosol [2,3,31,32,33]. Given the expression profile and subcellular localization of BnABH205, we embarked on an in-depth investigation to assess how genetic diversity within this gene impacts SOC. Within the *BnABH205* coding sequence (CDS), 34 single-nucleotide polymorphisms (SNPs) were identified, along with an insertion–deletion (InDel) located in the 3′ untranslated region (UTR) (Table S12). According to a recent genome-wide association study by Tang et al. [32], five haplotypes of the gene corresponding to *BnABH205* exhibited a moderate correlation with SOC. These haplotypes encompassed nine SNPs resulting in missense variants in the CDS and one InDel situated in 3′ UTR regions (Figure 9B and Table S12). To visually scrutinize any structural variations among these haplotypes, we predicted the translations and illustrated the three-dimensional protein structures of five haplotypes that impacted that protein sequence (Hap_0 through Hap_4) of BnABH205 (Figure 9C). Comparison of these five protein structures revealed very low root-mean-square deviation values (RMSD ≤ 0.115), indicating that the amino acid alterations in these haplotypes had minimal effects on structural variations.

Haplotypes 3 and 4 encoded a mutation from tyrosine (Y) to phenylalanine (F) at position 78 (Figure S12 and Table S12). Using an established inbred population of *B. napus*, the SOC was assessed in the five haplotypes of BnABH205. We observed that germplasm carrying Hap_1 exhibited significantly higher SOC levels, whereas those with Hap_4 demonstrated reduced SOC accumulation (Figure 9D).

The accumulation of oil in rapeseed primarily occurs during the mid-to-late stages of development. Given the highly specific expression of *BnABH205* during this phase and its significant upregulation in response to heat stress (Figures S8 and S10), plants of cultivar ZS11 at the later stages of seed maturation were subjected to exposure to 40 °C heat. As displayed in Figure 9E–G, a notable reduction in SOC was observed following high-temperature exposure, whereas no substantial changes were detected in the composition of unsaturated or saturated fatty acids. These findings indicate that BnABH205 may regulate SOC under heat stress by modulating the overall rate of oil synthesis rather than altering the fatty acid composition.

## 3. Discussion

The SOC of *B. napus* is primarily regulated by the dynamic balance between TAG synthesis and breakdown during seed maturation. This process involves precise control of FA synthesis, TAG assembly, and TAG encapsulation within oil bodies [3,4,5,31,32,33,34]. A crucial aspect of this intricate biochemical pathway is the formation of TAG in the plastid and ER, which is largely influenced by the activity of esterases and lipases [3,4,5,31,32,33,34]. Plants possess a diverse array of esterases/lipases, with ABHs being a notable example [10]. ABHs are widespread across almost all living organisms and exhibit a broad substrate specificity, playing roles in plant growth modulation through both catalytic and non-catalytic functions [10]. Despite their importance, to date, only a limited number of ABH family members have been characterized in plant species [10]. Recently, the availability of a high-quality genome sequence and comprehensive transcriptome datasets for the *B. napus* cultivar ZS11 has enabled the identification and expression profiling of genes such as the *BnABHs* [28,35,36].

In the current study, we identified 777 *ABH*-like genes in the *B. napus* cultivar ZS11, marking a roughly 1.2-fold increase compared to the number of *AtABHs* previously reported [10]. Unexpectedly, the count of *BnABHs* did not significantly exceed the numbers initially reported in *A. thaliana*, which contrasts with earlier studies [6,28,29,30,35,36]. A prior genome-wide analysis of the *ABH* superfamily in *A. thaliana* had reported 638 members [10]. To address this discrepancy, we reevaluated the AtABH superfamily using the HMMER model, NCBI CDD online tool (https://www.ncbi.nlm.nih.gov/Structure/bwrpsb/bwrpsb.cgi accessed on 14 April 2023) and SMART Tool (https://smart.embl.de/ accessed on 25 April 2023). This comprehensive reassessment confirmed the presence of 265 members in the *AtABH* superfamily (Table S2).

Based on phylogenetic analysis, we categorized BnABHs into 10 groups (C1 to C10, Figure 1 and Table S2). The distribution of BnABH proteins among these phylogenetic clades was uneven (Figure 1), with clade C6 accounting for approximately 20% of the total (Figure 1 and Table S2). This suggests potential diversification of clade C6 within the *B. napus* genome. Furthermore, the variation observed in amino acid residue count, isoelectric point (pI), and predicted subcellular localization of BnABH proteins (Table S1) may indicate their functional diversity in various biological processes. This functional versatility could be attributed to differences in the full-length amino acid sequences or domains beyond the conserved α/β-hydrolase fold domain.

ABHs exhibit broad substrate specificity and are capable of catabolizing a wide range of lipids, making their biochemical activity readily detectable [10,12]. Through mass spectrometry analysis, we identified 24 BnABHs in region II of the esterase isoenzyme zymogram (Figure 4), a crucial identification of potential ABH-type esterases/lipases involved in oil accumulation during seed maturation. Examination of the motifs, gene expression profiles, and *cis*-regulatory elements of the C3 group *BnABHs* hints at the roles of these members in regulating SOC throughout seed development and in response to various environmental stresses (Figure 5, Figure 6, Figure 7, Figure 8 and Figure 9). Notably, our mass spectrometry analysis also detected the presence of other esterases/lipases beyond ABH-type proteins (Figure 4B), consistent with previous reports [6,8]. These include GDSL-type esterases/lipases and serine carboxypeptidases, suggesting that additional esterases/lipases may also contribute to lipid accumulation during seed maturation.

A thorough analysis revealed the presence of 12 *cis*-acting elements within the promoter regions of *BnABH* genes belonging to the C3 group (Figure 8 and Figure S11, and Tables S10 and S11), suggesting that the expression of these genes may be modulated by ABA, hypoxia, MeJA, and cold stress. Notably, a substantial majority of the *BnABH* genes in the C3 group (63 out of 69) contain ABA-responsive elements. During seed maturation, dehydration in the siliques likely triggers the accumulation of ABA, which in turn may regulate the expression of genes involved in seed oil accumulation. These genes include those responsible for lipid metabolism and for preventing premature seed germination within the pods. However, the complex interaction between ABA accumulation during seed dehydration and oil accumulation warrants further investigation.

SOC is a multifaceted characteristic shaped by a mix of genetic and environmental factors, with seed development occurring under a range of climatic conditions and abiotic stressors [31,32]. Temperature exerts a significant effect on both the SOC and FA composition in plants [24,25,26,27]. Specifically, SOC tends to decrease markedly as temperature increases during seed maturation, whereas it is generally elevated at lower growth temperatures [24,25,26,27]. By employing quantitative genetics and omics approaches like QTL mapping and genome-wide association studies (GWASs), researchers have identified an increasing number of quantitative trait loci (QTLs) and quantitative trait nucleotides (QTNs) linked to SOC in rapeseed [2,3,5,31,32,33,37]. Our findings further revealed that *BnABH205* exhibits high expression levels during seed maturation (Figure S8). This elevated expression is potentially associated with the abundance of promoter elements responsive to plant growth and development identified within its promoter regions (Figure 8). Comparison of three-dimensional structures of BnABH205 and five identified haplotypes of BnABH205 indicated a striking level of structural similarity to their AtABH homologs (Figure 9, Figures S5 and S6). This similarity aligns with the distribution of conserved ABH motifs across these proteins. Additionally, the localization of BnABH205 to the plastid (Figure 9A) suggested that BnABH205 functions as an active esterase, playing a pivotal role in TAG assembly within the plastid during seed maturation.

## 4. Materials and Methods

### 4.1. Identification of the ABH Gene Family in Brassica napus

Genome resources for *B. napus* (cultivar ZS11.v10) were sourced from the BnTIR database (https://yanglab.hzau.edu.cn/bntir accessed on 15 March 2023) [36]. A total of 638 previously characterized alpha/beta-hydrolase proteins (ABHs) from *A. thaliana* [10] were retrieved from the TAIR database (http://www.arabidopsis.org/ accessed on 15 March 2023) using specific sequence accession numbers as queries. To identify *ABH* genes within the rapeseed genome, a BlastP search was performed using Blast-2.14.0.1+ software, with an E-value threshold of 10^−10^. Subsequently, the Hidden Markov Model (HMM) profile of the ABH domain, encompassing PF12697, PF00561, PF07859, PF12695, PF06259, and PF12715, was downloaded from the InterPro database (https://www.ebi.ac.uk/interpro/ accessed on 20 March 2023). HMMER3.0 software was then employed to query the *B. napus* protein sequence database, again using10^−10^. The resulting data were merged and deduplicated, and the identified domains were validated using both the NCBI Batch CD-Search tool (https://www.ncbi.nlm.nih.gov/Structure/cdd/wrpsb.cgi accessed on 14 April 2023) and the SMART Tool (http://smart.emblheidelberg.de/ accessed on 25 April 2023), as previously outlined [6,29,30]. Following this validation process, we successfully identified all candidate genes encoding the ABH domain in the genomes of *B. napus* (comprising 777 members) and *A. thaliana* (comprising 265 members), respectively (Tables S1 and S2).

### 4.2. Analysis of Predicted ABH Proteins

Full-length ABH proteins from *B. napus* and *A. thaliana* were subjected to multiple sequence alignments using the FFT-NS-I approach provided by MAFFT v7.490 software [38]. For phylogenetic tree construction, the maximum likelihood method implemented in FastTree v2.1.11 was employed [39]. The phylogenetic tree was visualized using FigTree v1.4.4 [6,29,30]. EXPASY (https://web.expasy.org/protparam/ accessed on 17 February 2024) was utilized to calculate the molecular weight, theoretical isoelectric point (pI), and instability index of members of the BnABH protein family. Subcellular localization of BnABH proteins was predicted using Wolfpsort (https://wolfpsort.hgc.jp/ accessed on 20 February 2024). The MEME suite (https://meme-suite.org/meme/tools/meme accessed on 21 February 2024) was employed to identify conserved motifs within BnABH proteins, with the analysis limited to a maximum of 10 motifs.

### 4.3. Genome-Scale Synteny Analysis of ABH Genes

The genomic sequence assembly and annotation datasets for *B. napus* cultivar ZS11 and *A. thaliana* were obtained from the BnTIR database (https://yanglab.hzau.edu.cn/bntir accessed on 15 March 2023) and the TAIR repository (http://www.arabidopsis.org/ accessed on 15 March 2023), respectively. To perform and visually represent the synteny analysis, we utilized the One Step MCScanX plugin (with an E-value cutoff set at 10^−10^) and the Advanced Circos plugin within the TBtools v2.210 program [40]. This analysis focused on the *BnABH* family genes, as well as their relationships with *A. thaliana*, as previously described [6,29,30,40].

### 4.4. Chromosome Localization and Gene Structure Analyses

The chromosomal locations of the *BnABH* genes were determined using genomic annotation data from the *B. napus* cultivar ZS11 genome, obtained from BRAD (http://www.brassicadb.cn/ accessed on 23 February 2024) [41]. Chromosome mapping was performed and visualized using TBtools v2.210 [40]. A GFF3 file from the *B. napus* cultivar ZS11 genome provided detailed information on gene structures, enabling the identification of exons and introns within the *BnABH* genes. Gene configurations were visualized using TBtools v2.210 [6,29,30,40].

### 4.5. Expression Profiles of BnABH Genes Determined by Transcriptome Data

Publicly available RNA-sequencing (RNA-seq) datasets from 12 tissues (root, stem, leaf, flower, silique, sepal, pistil, stamen, ovule, pericarp, wilting pistil, and blossomy pistil) of *B. napus* cultivar ZS11 were obtained from the NCBI database (project ID: PRJNA394926). Additionally, RNA-seq data pertaining to various stress conditions (dehydration, NaCl, ABA, and cold conditions) were retrieved from the NGDC database (project ID: CRA001775) [6,29,30]. The quality of each dataset was evaluated using FastQC_v0.12.1, after which adapter trimming and quality filtering were performed using Trimmomatic_0.39. The transcriptome reads were then mapped to the *B. napus* cultivar ZS11 reference genome using Hisat2_2.04. Transcript assembly and quantification were conducted using the StringTie plugin within TBtools v2.210, resulting in TPM (transcripts per kilobase million) expression levels. The Log_2_-normalized expression data were visually represented as heatmaps using TBtools v2.210, as previously described [6,29,30,40].

### 4.6. Identification of Cis-Elements in the Promoters of the Clade-C3 BnABH Genes

For each *ABH* gene within clade C3, 2000-base pair (bp) sequences upstream of the coding sequence (CDS) were isolated from the *B. napus* cultivar ZS11 genome and designated as the promoter sequence. PlantCARE was employed to predict the presence of *cis*-regulatory elements (http://bioinformatics.psb.ugent.be/webtools/plantcare/html/ accessed on 9 March 2025) [42]. Following the categorization system introduced by Mengarelli et al. (2021), the predicted *cis*-acting elements were classified into three categories: abiotic and biotic stresses, phytohormone response, and plant growth and development [43]. Core promoter elements (TATA-box, CAAT-box) and uncategorized boxes were excluded from the analysis. The data were subsequently subjected to classification and statistical analysis. Visualization was achieved through the generation of a heatmap and stacked bar charts using the heatmap v4.4.2 and ggplot2 v3.5.1 packages, respectively, as previously described [6,29,30,40].

### 4.7. Plant Growth, Esterase Isozyme Electrophoresis, and Mass Spectrometry Analysis

The *B. napus* cultivar ZS11 was employed in this study. Initially, seeds of this cultivar were grown in a greenhouse at 24 °C with a 14/10 h day/night cycle. For vernalization, plants of the *B. napus* cultivar ZS11 at the six-leaf stage were subjected to cold treatment at 4 °C with an 8/16 h day/night cycle. Following vernalization, the plants were reverted to the original greenhouse conditions of 24 °C with a 14/10 h day/night cycle. Silique samples were collected at 4, 34, 38, 42, 46, 50, 54, and 58 DAF (Days After Fertilization) and subsequently frozen at −80 °C for esterase isozyme electrophoresis analysis. During the final stage of seed maturation, which began at 58 DAF, the plants were exposed to a 14/10 h day/night cycle with temperatures of 40/30 °C (day/night), respectively, for durations ranging from 1 to 4 days. Upon completion of these treatments, ten siliques were harvested from each plant and stored at −80 °C for further esterase isozyme analysis. A 0.2 g specimen was accurately measured and ground in a mortar with 2 mL of pre-cooled Tris-HCl extraction buffer (pH 8.0). The homogenate was then centrifuged at 20,000 rpm for 10 min at 4 °C, and the resulting supernatant was collected for further examination. Protein content was determined using the bicinchoninic acid (BCA) assay, with absorbance readings taken at 570 nm on a microplate reader. Subsequently, 200 ng of the extracted protein was separated by polyacrylamide gel electrophoresis using an 8% resolving gel and a 3.6% stacking gel. The entire electrophoresis process was continuously performed at 4 °C under a constant current of 20 mA. Following electrophoresis, the gel was stained with an esterase visualization solution consisting of 1% α- naphthyl acetate, 2% β-naphthyl in an ethanol solution, and 2% naphtanil diazo blue B dissolved in 50 mM phosphate buffer at pH 6.4. Distinct esterase isozyme bands visible on the zymogram were excised for identification via mass spectrometry, which was carried out following previously established protocols [6].

### 4.8. Three-Dimensional Structure Simulation and Subcellular Localization Analysis

The three-dimensional structures of BnABH205 paralogs and AtABH proteins were predicted using the Alphafold3 online tool (https://alphafoldserver.com/ accessed on 22 March 2025) [44]. Subsequently, these protein architectures were visualized with PyMOL version 3.0.3.

To generate GFP-labeled versions of BnABH205, the coding sequence (CDS) lacking its termination codon (Table S13) was inserted into the Cam-35S-GFP vector between the *Bam*HI and *Sal*I restriction sites. This resulted in a C-terminal GFP fusion designated as *35S:BnABH205-GFP*. After successful sequence verification, the recombinant plasmids were introduced into *Agrobacterium tumefaciens* strain GV3101 and infiltrated into the lower surface of 6-week-old *Nicotiana benthamiana* leaves for transient protein expression. Following 72 h of routine cultivation, GFP fluorescence signals were observed using Laser Scanning Confocal Microscopy (LSCM) (FV 3000, Olympus, Tokyo, Japan) at an excitation wavelength of 488 nm [45,46].

### 4.9. Analysis of Variations in BnABH205 and Assessment of Seed Oil Content

Single-nucleotide polymorphisms (SNPs) within the *BnABH205* gene were retrieved from the *B. napus* database (BnIR: https://yanglab.hzau.edu.cn/ accessed on 4 April 2025) [36]. Ten SNPs were selected with a polymorphism information content (PIC) exceeding 0.25 and that would cause missense amino acid substitutions. Utilizing the multi-locus tool provided on BnIR, we generated potential haplotype combinations. Subsequently, low-frequency haplotypes with a minor allele frequency (MAF) below 0.05 were eliminated. This refinement process resulted in the retention of five primary haplotypes (Hap_0 through Hap_4), with population frequencies ranging from 0.059 to 0.287. We conducted an association study between seed oil content and these gene haplotypes using data from a natural *B. napus* population established by Tang et al. [32]. Seed oil content (SOC) and fatty acid composition were determined with near-infrared spectroscopy (ANTARIS II, Thermo Scientific™, Waltham, MA, USA) as previously described [2,6]. Statistical analysis, encompassing error analysis and significance testing, was conducted using GraphPad Prism version 9.5.1. For column analyses, a one-way analysis of variance (ANOVA) model was employed to determine statistical significance. Each experiment was repeated at least three times to ensure reliability. Error bars represent the standard deviation, and asterisks (* *p* < 0.05) are used to indicate statistically significant differences.

## 5. Conclusions

In the current study, we performed a comprehensive genome-wide analysis of the α-/β-hydrolase family in the *Brassica napus* cultivar ZS11. Employing rigorous bioinformatics methodologies, we successfully identified a total of 777 *BnABH* genes, marking a significant expansion of the *ABH* gene family compared to the re-assessed family in *Arabidopsis thaliana*. Of particular interest was the identification of 24 BnABH proteins that were specifically active during seed maturation. Based on phylogenetic analysis, most of these proteins (11 out of 24) were classified into clade C3. The gene and protein structures, expression patterns, and *cis*-elements of the C3 group of *BnABH* genes were further analyzed. Further investigation of *BnABH205* through three-dimensional structural modeling, subcellular localization, and correlation of *BnABH205* expression with SOC confirmed its role as an active esterase localized to the plastid that plays a crucial role in TAG assembly during seed maturation in *B. napus*. This discovery not only enhances our understanding of the ABH superfamily in rapeseed but also reveals new avenues for future research aimed at elucidating the specific mechanisms by which BnABH205 and other C3-group BnABH proteins contribute to seed oil metabolism and accumulation.

## Data Availability

Data is contained within the article.

## Supplementary Materials

The following supporting information can be downloaded at: https://www.mdpi.com/article/10.3390/ijms26104746/s1.
