# Supplementary material for: The Alpha/Beta-Hydrolase Fold Superfamily in Brassica napus: Expression Profiles and Functional Implications of Clade-3 BnABH Proteins in Response to Abiotic Stress
Source: Int J Mol Sci. 2025 May 15;26(10):4746. doi: 10.3390/ijms26104746 (PMC12112387; doi:10.3390/ijms26104746)
Supplement: Supplementary file 1 [file ijms-26-04746-s001.zip › Figure S1-12.pdf]

Figure S1

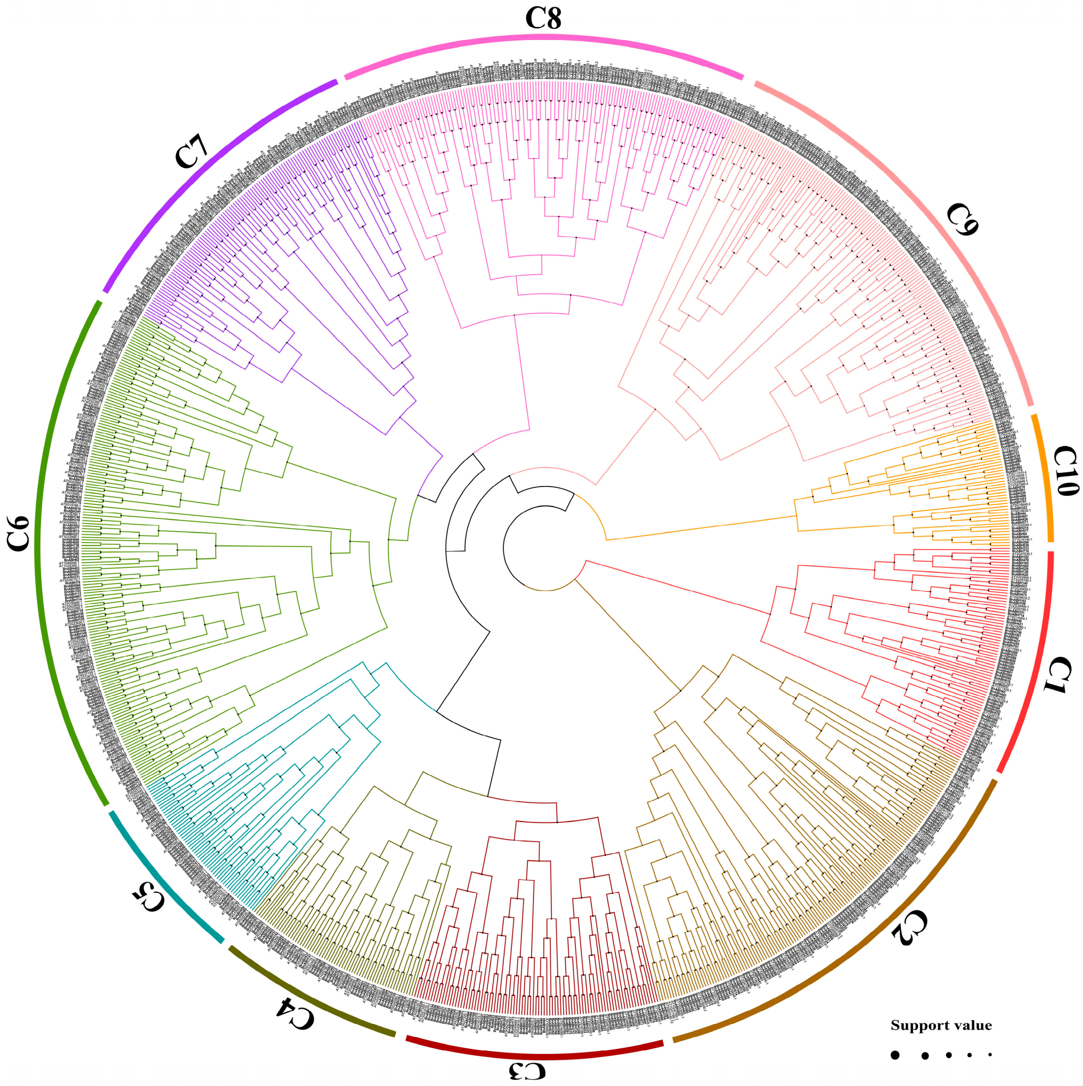

Figure S1. Phylogenetic analysis of the AtABH and BnABH proteins.

These proteins were clustered into 10 subclades, and the branches corresponding to different subclades were marked with different colors. The gene IDs are displayed at the periphery of the phylogenetic tree. The size of the black dot indicates the nodal support.

Figure S2

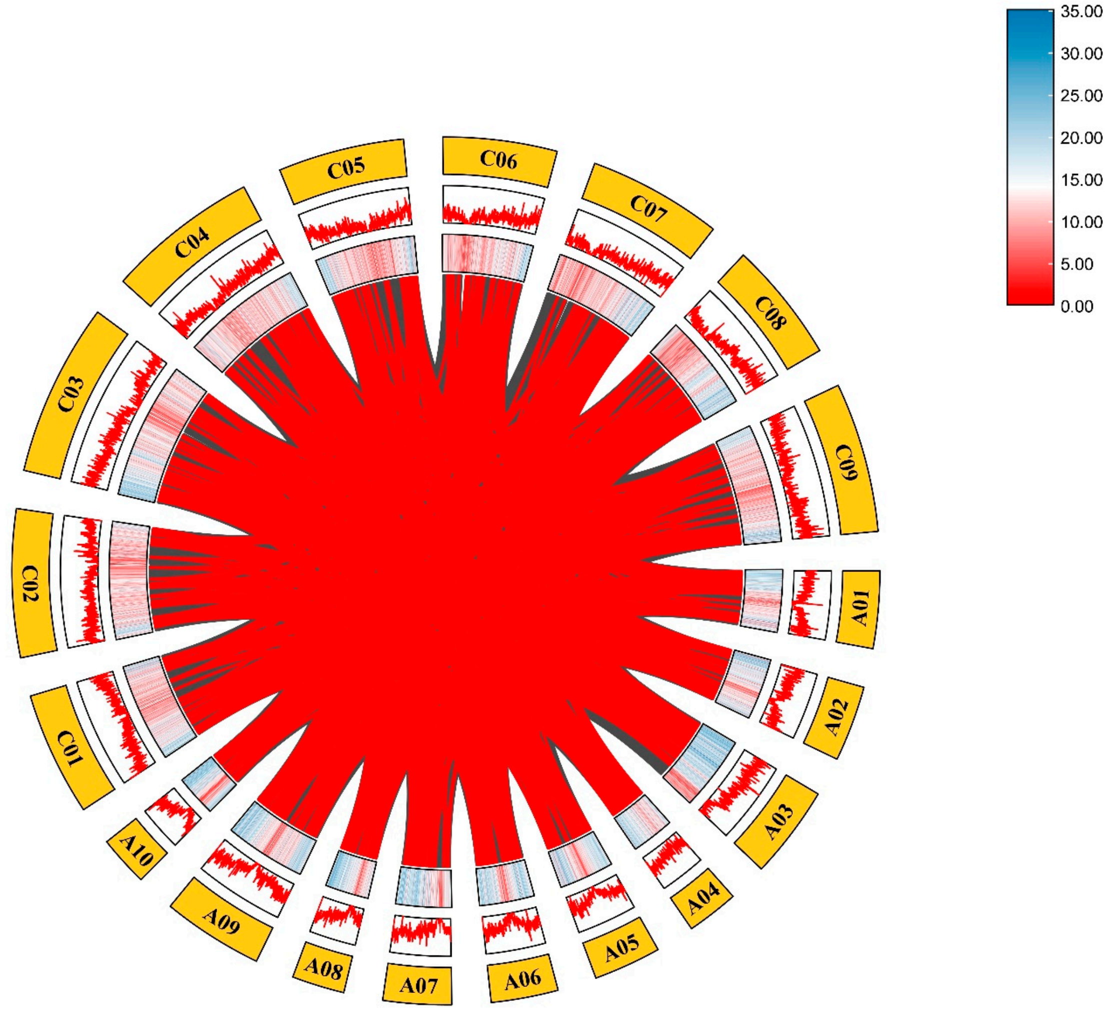

Figure S2. The synteny analysis of *BnABH* genes in *B. napus*.

The gray lines represent all synteny blocks, while the red lines highlight segmental duplicate gene pairs within the *B. napus* cultivar ZS11 genome. Gene density is visualized as a heatmap and line plot outside the circular ideogram.

Figure S3

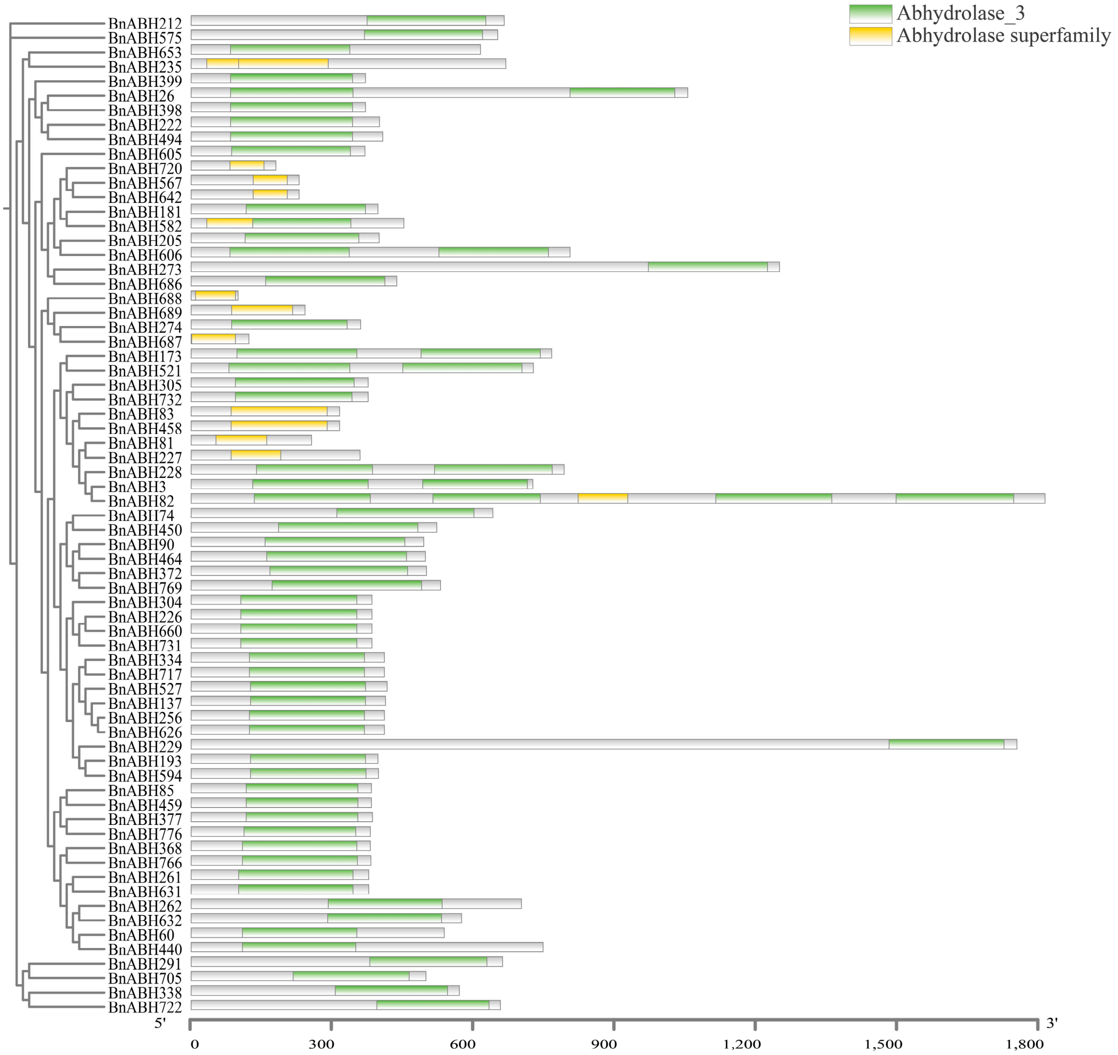

Figure S3. The conserved ABH domain of BnABH proteins in clade 3.

The conserved ABH domains are indicated with green and yellow boxes.

Figure S4.

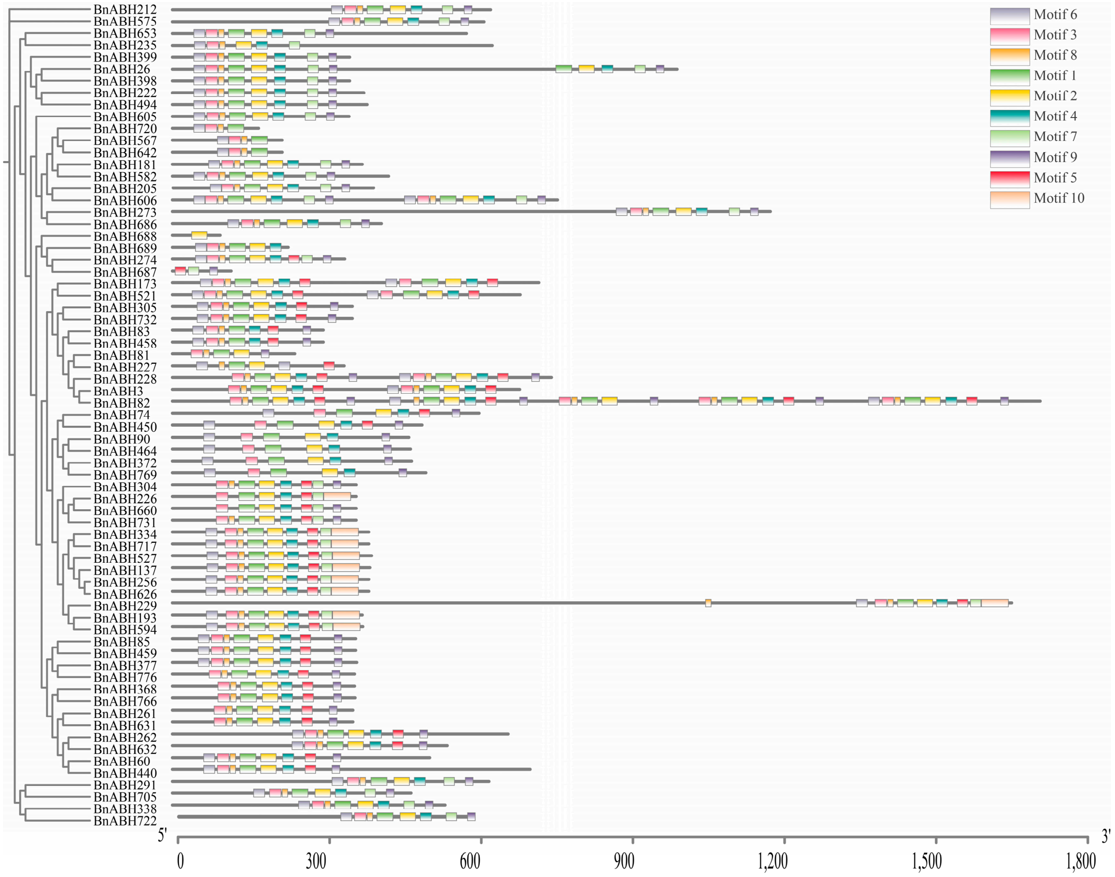

Figure S4. Conserved motifs of BnABH proteins in clade 3.

Boxes of different colors represent different motifs.

Figure S5

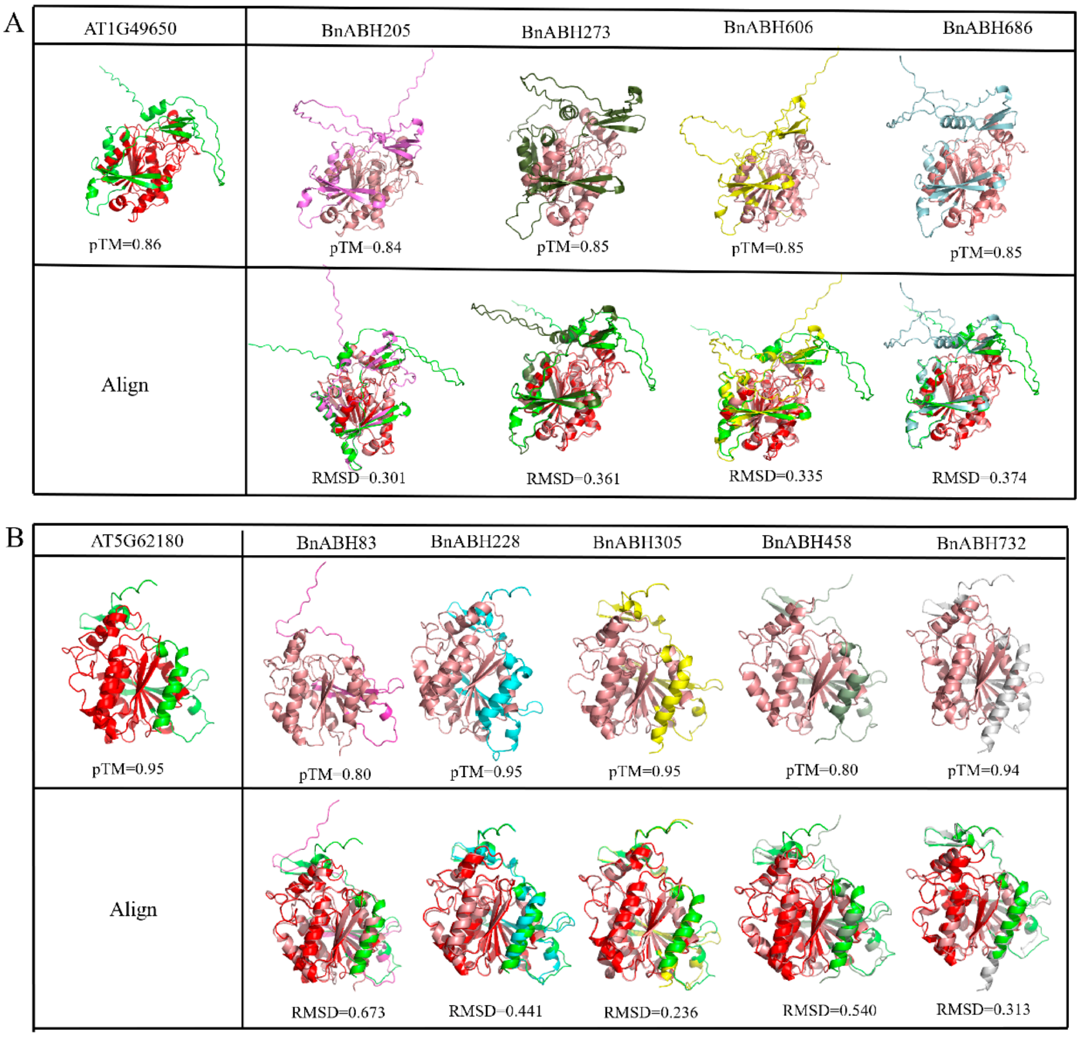

Figure S5. The three-dimensional structure of 11 ABH proteins in the clade 3.

(A) 3D structure of AT1G49650 and its *B. napus* homologous proteins. (B) 3D structure of AT1G49650 and its *B. napus* homologous proteins. The ABH domain of *A. thaliana* is colored red, while *B. napus* ABH domain is shown in pink. Non-ABH structural regions are labeled with distinct colors across different proteins. The pTM values predicted by AlphaFold3 for each protein are labeled below the corresponding protein. The alignment was performed using PyMOL 3.0.3 software, and the RMSD values obtained from the alignment were marked below the corresponding proteins.

Figure S6

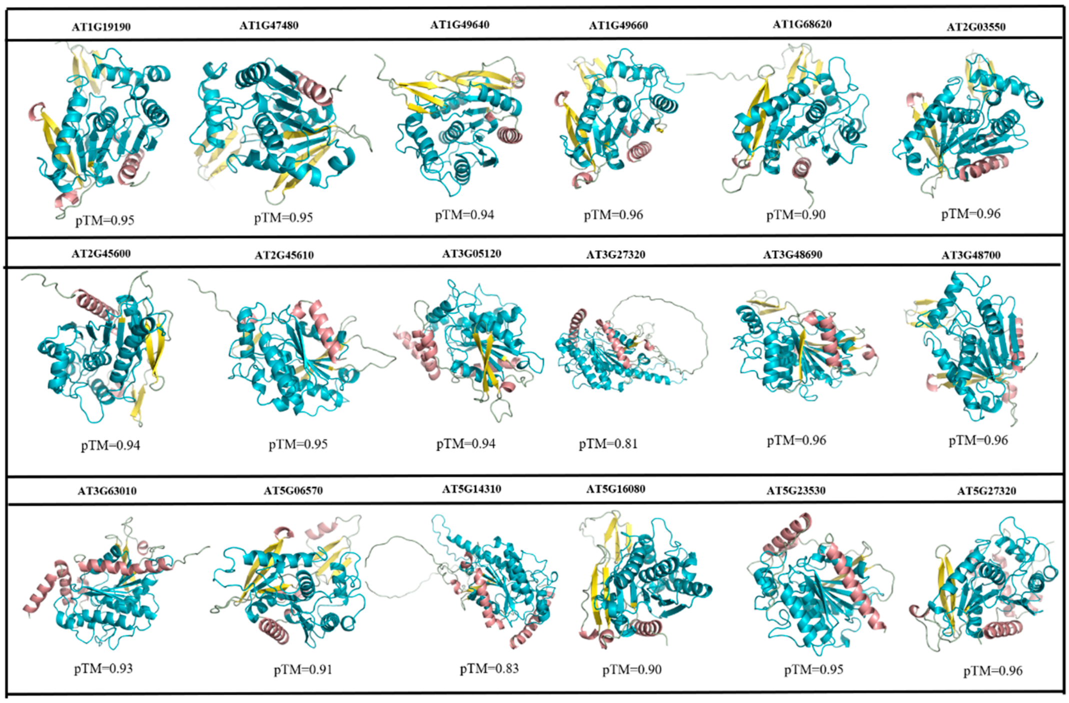

Figure S6. The three-dimensional structure of 18 *A. thaliana* ABH proteins in the clade 3.

Prediction of 18 *A. thaliana* ABH proteins tertiary structure. Helix, sheet, and loop structure are labeled with pink, yellow, and cyan respectively, and the ABH domain is presented in the blue region. The pTM values predicted by AlphaFold3 for each protein are labeled below the corresponding protein.

Figure S7.

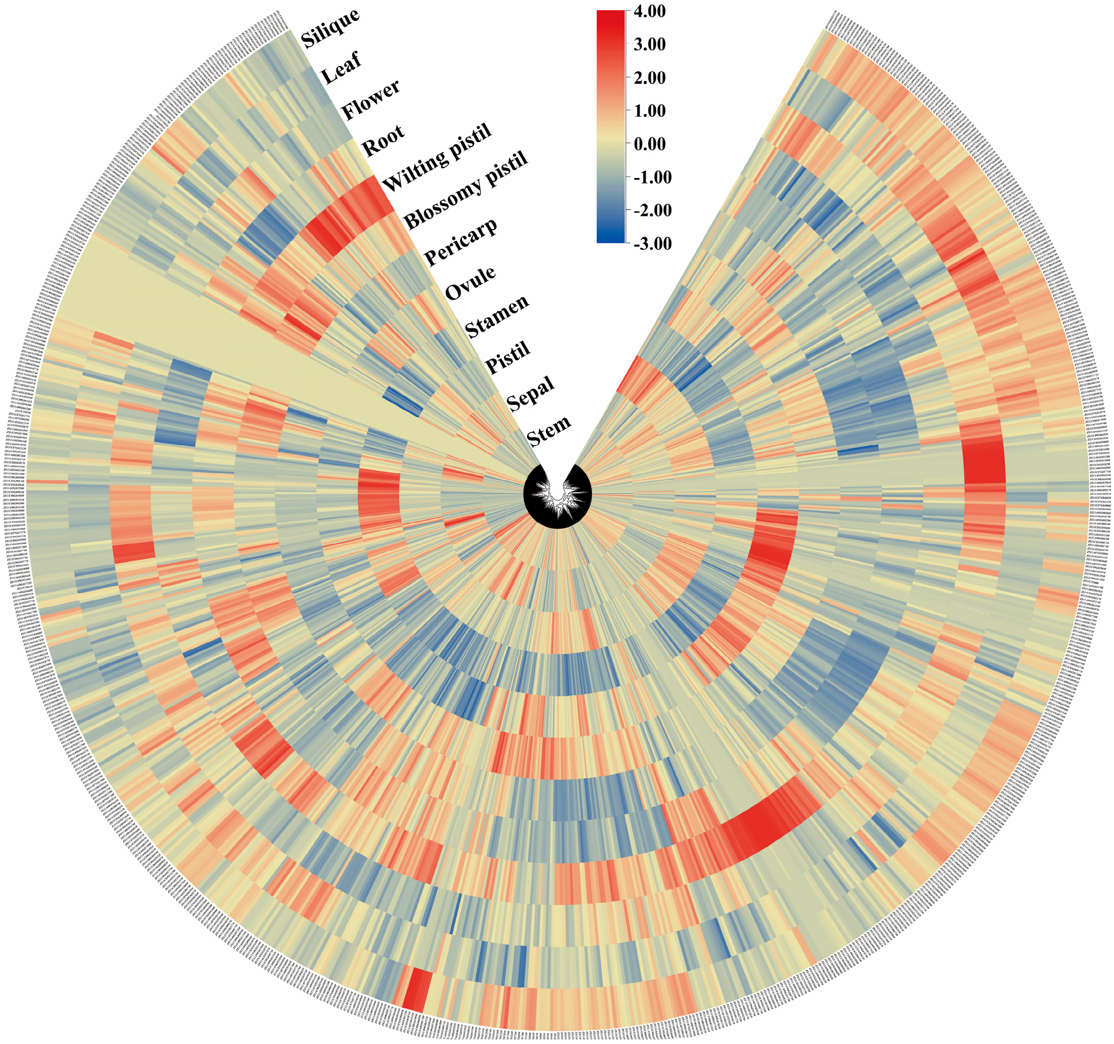

Figure S7. Expression profiles of the *BnABH* genes in different tissues.

The expression level is equal to the mean values and transforms log_2 values for normalization. The color scale represents relative expression levels from low (blue colored) to high (red colored).

Figure S8.

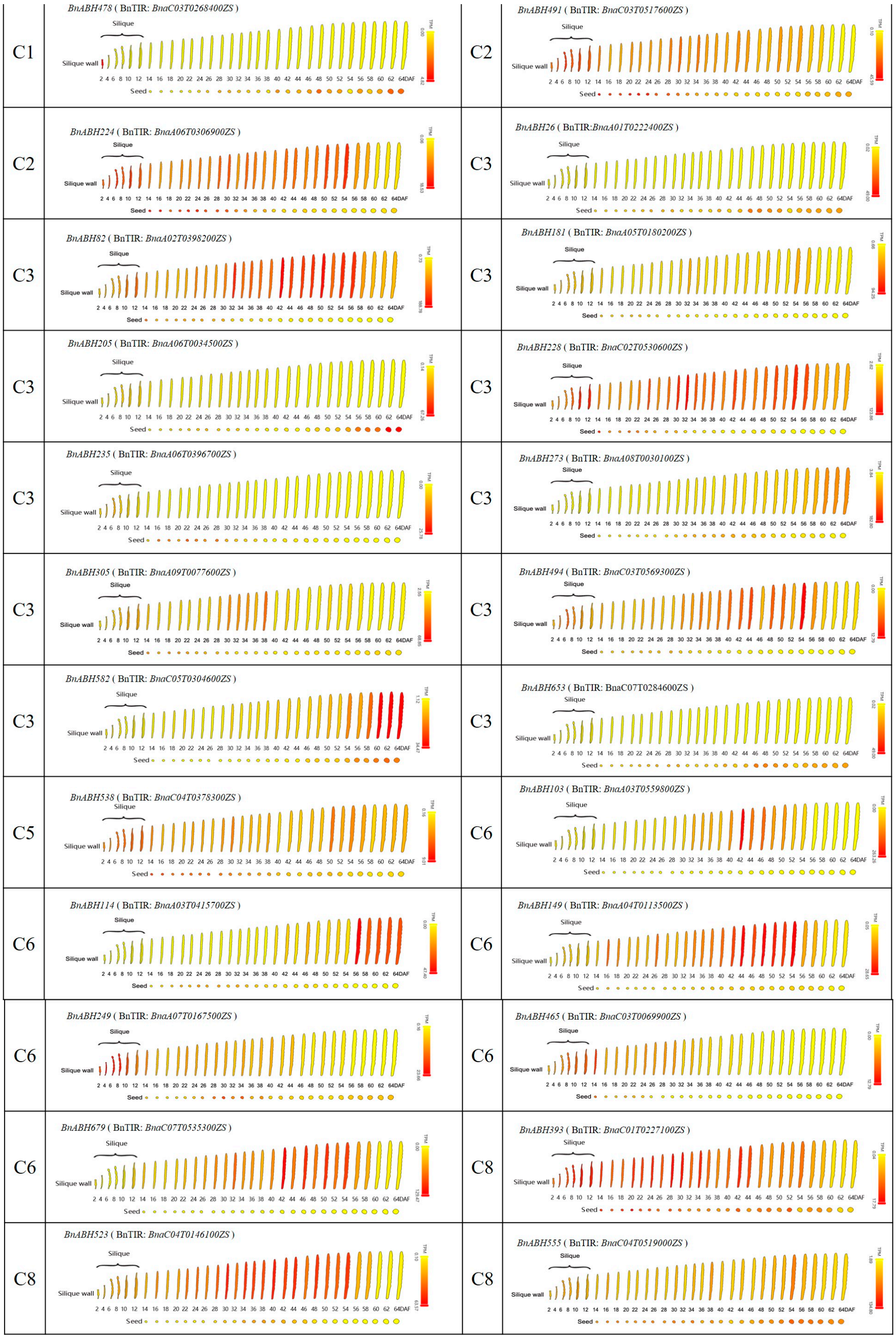

Figure S8. The expression level of the identified *BnABH* genes in the seeds.

The expression level of the identified *BnABH* genes in the seeds were obtained from the *B. napus* RNA-seq data (http://yanglab.hzau.edu.cn/BnTIR). The color scale represents relative expression levels from low (yellow colored) to high (red colored).

Figure S9.

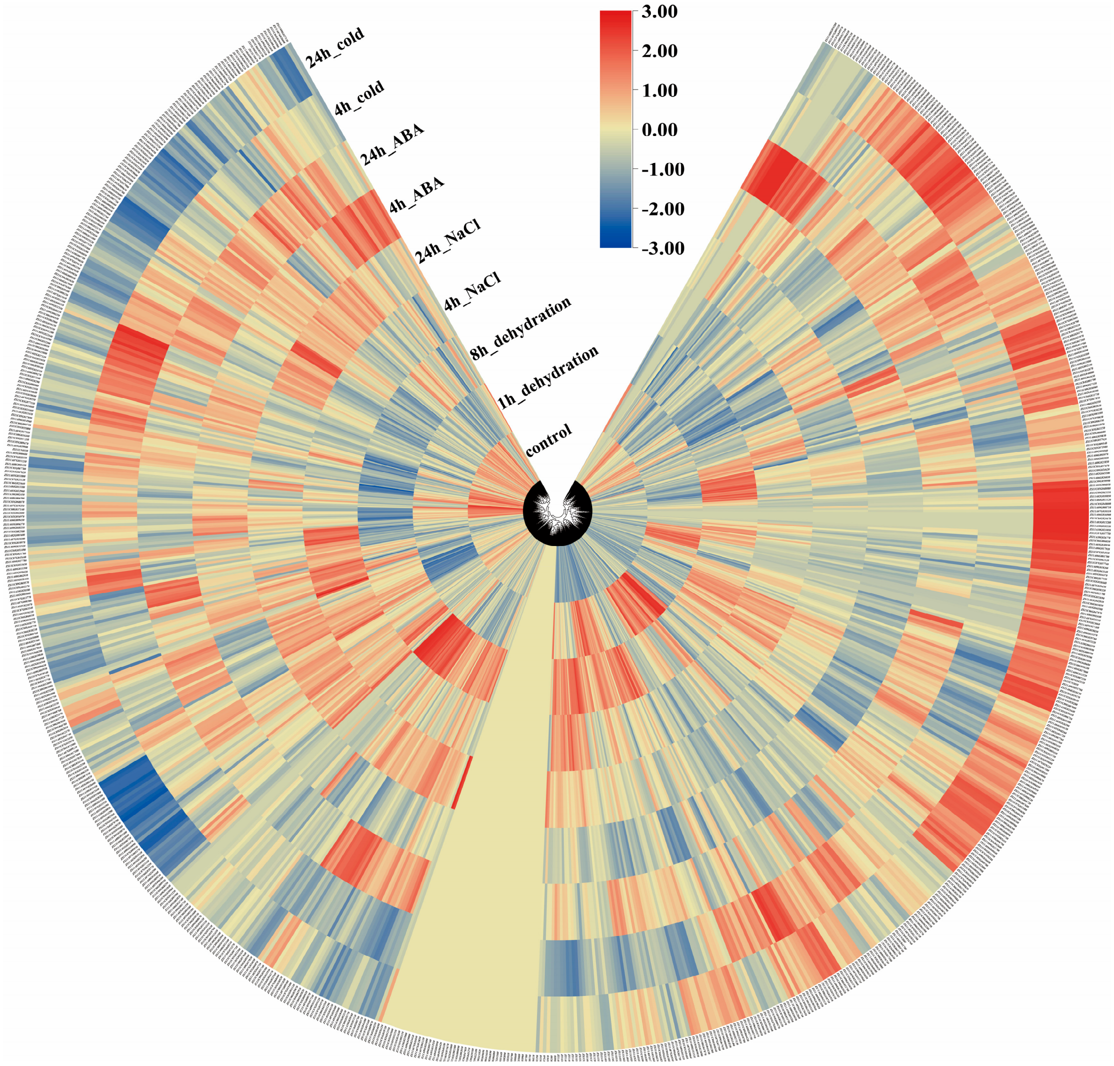

Figure S9. Expression profiles of the *BnABH* genes under different stresses.

The expression level is equal to the mean values and transforms $\log_2$ values for normalization. The color scale represents relative expression levels from low (blue colored) to high (red colored).

Figure S10

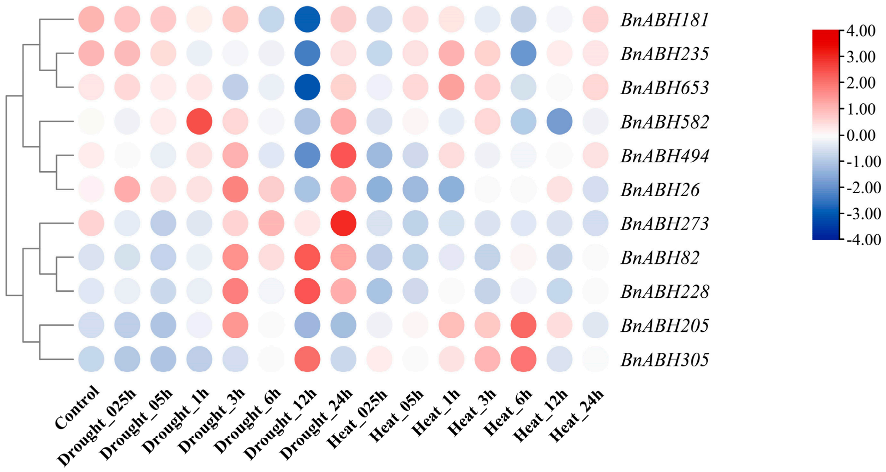

Figure S10. Gene expression profiles of *ABH* genes in the C3 clade identified by mass spectrometry under heat stress and drought stress conditions.

The expression data under drought and heat stress are sourced from the rapeseed database BnIR (https://yanglab.hzau.edu.cn/BnIR).The ordinate lists the names of relevant ABH genes. The color scale represents relative expression levels from low (blue colored) to high (red colored).

Figure S11

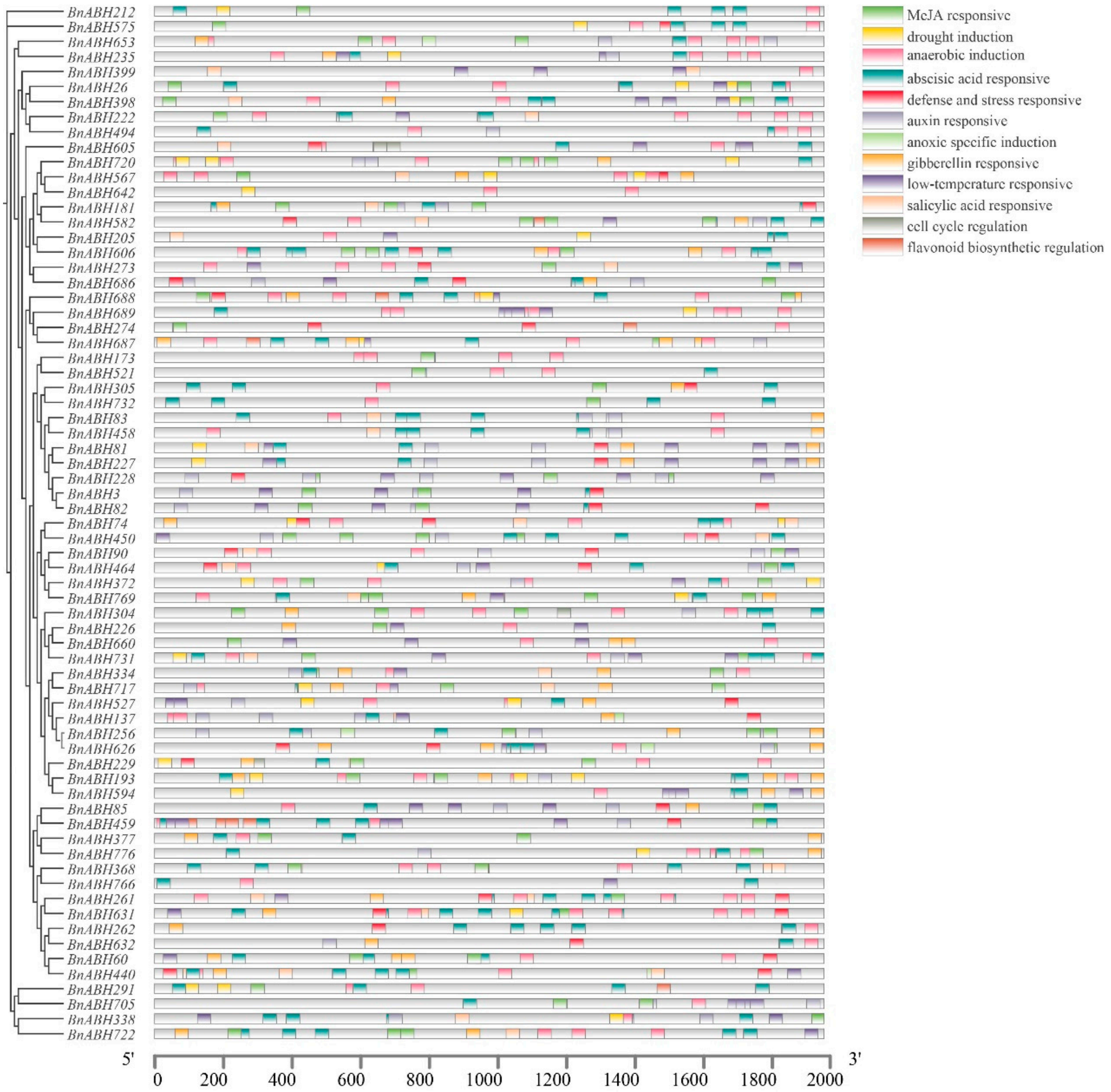

Figure S11. *Cis*-elements found in the promoter region of the clade 3 *BnABH* genes.

The name of each regulatory element is shown on the right panel, with different colored boxes representing different *cis*-elements. The relative location of each element on each promoter can be estimated by the scale at the bottom.

Figure S12

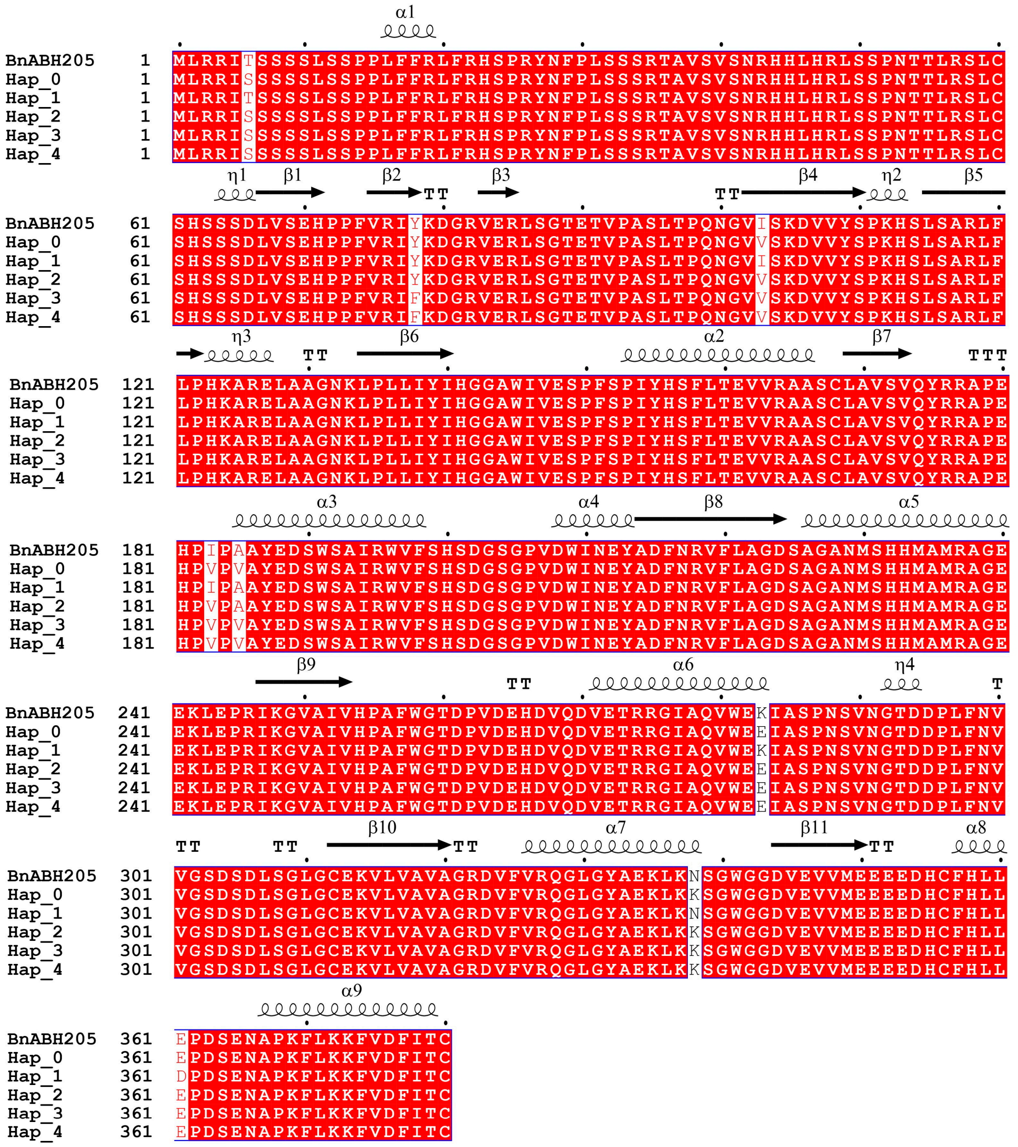

Figure S12. Sequence alignment of BnABH205 and its haplotype (Hap_0 - Hap_4) proteins. Amino acid sites with the same residue are shown on a red background; sites with different amino acids are marked by colored boxes. Secondary structure elements of the proteins, including α - helices (α) and β - sheets (β), are labeled above.
